# Using Goal-Directed Design to Create a Mobile Health App to Improve Patient Compliance With Hypertension Self-Management: Development and Deployment

**DOI:** 10.2196/14466

**Published:** 2020-02-25

**Authors:** Huilong Duan, Zheyu Wang, Yumeng Ji, Li Ma, Fang Liu, Mingwei Chi, Ning Deng, Jiye An

**Affiliations:** 1 College of Biomedical Engineering and Instrument Science Ministry of Education Key Laboratory of Biomedical Engineering Zhejiang University Hangzhou China; 2 General Hospital of Ningxia Medical University Yinchuan China

**Keywords:** goal-directed design, smartphone, mobile health, patients, hypertension self-management, mobile phone

## Abstract

**Background:**

Hypertension is a lifestyle-induced chronic disease that threatens the lives of patients. Control of hypertension requires patients to follow self-management regimes; unfortunately, however, patient compliance with hypertension self-management is low, especially in developing countries. Improvement of patient compliance is premised on meeting patient needs. Mobile health apps are becoming increasingly popular for self-management of chronic diseases. However, few mobile apps have been designed to meet patient needs for hypertension self-management.

**Objective:**

The goal of this study was to develop a mobile health app to improve patient compliance with hypertension self-management and evaluate the effectiveness of the app in terms of patient compliance.

**Methods:**

The goal-directed design method was applied to guide study design. We divided the study into 4 stages. Stages 1 to 3 comprised the development process. To improve the applicability of the goal-directed design method to chronic disease management, we extracted elements of user models concerned with patient compliance and defined a concrete process for user modeling. In stage 1, personas of hypertensive patients were built using qualitative and quantitative methods. Clustering methods based on questionnaire responses were used to group patients. Qualitative interviews were conducted to identify the needs of different groups. In stage 2, several functional modules were designed to meet the needs of different groups based on the results from stage 1. In stage 3, prototypes of functional modules were designed and implemented as a real app. Stage 4 was the deployment process, in which we conducted a pilot study to investigate patient compliance after using the app. Patient compliance was calculated through the frequency with which they took blood pressure measurements. In addition, qualitative interviews were conducted to learn the underlying reasons for the compliance results.

**Results:**

In stage 1, patients were divided into 3 groups based on 82 valid questionnaire responses. Eighteen patients from the different groups (7, 5, and 6 patients) were interviewed, and the needs of the groups were summarized as follows: improve self-management ability, enhance self-management motivation, and receive self-management support. In stages 2 and 3, 6 functional modules were designed and implemented based on specified needs, and the usability of the app was improved through usability tests. In stage 4, 143 patients were recruited to use different versions of the app for 2 months. Results show that patient compliance improved as functional modules were added (*P*<.001) and was maintained at a high level (rate of 0.73). Interview results from 32 patients show that the design of the app met different needs; thus, patients were more compliant with it.

**Conclusions:**

This study developed a mobile health app for hypertension self-management using the goal-directed design method. The app proved to be effective for improving patient compliance with hypertension self-management.

## Introduction

### Background

Chronic diseases are among the most prevalent and costly conditions worldwide [1]. Cardiovascular disease, cancer, chronic lung disease, and diabetes are four major chronic diseases that result in approximately 60% of global deaths [2], and hypertension is a leading cause of cardiovascular disease [3]. In China, more than 20% of the adults have hypertension, and more than 40% have prehypertension [4]. To improve the survival rate and life quality of hypertensive patients, long-term self-management along with supervision and intervention from doctors are essential [5]. Concretely, patients are required to take their medications as prescribed, change their behaviors to achieve a healthy lifestyle, and stick to a continuous self-monitoring regime of blood pressure (BP) measurements [6]. However, in practice, one significant obstacle is that patients do not always comply with the medical instructions for controlling their disease [7-9]. The World Health Organization has reported on the issue of noncompliance in treating prevalent chronic diseases such as hypertension, asthma, and diabetes [10]. Insufficient compliance may result in decreased treatment effectiveness or even treatment failure, which poses a great threat to health and life [11-13]. Although patients’ awareness of the need for compliance has increased in recent years, much work remains to improve compliance in hypertensive patients [14-16].

The rapid development of mobile technologies and the popularity of smartphones fostered the development of an enormous number of mobile health (mHealth) apps. Such apps have the potential to improve patient compliance with chronic disease management [17]. For example, mHealth apps can provide personalized self-management strategies to users, allowing them to avoid the time and resource burdens imposed by the frequent interventions provided by health care providers [18]. In terms of hypertension, smartphone technology has been shown to help patients improve BP control [19] and medication compliance [20], mainly through functional modules such as home BP monitoring, medication reminders, and health education. Concretely, patients can use their smartphones to record measured BP, receive reminders to take medications in the form of notifications or short messages, and acquire health knowledge about hypertension. However, in practice, patients have various goals and need to use mHealth apps to self-manage their conditions [21]. Nonetheless, the functional designs of mHealth apps sometimes fail to meet patient needs, which results in reduced patient motivation for using these apps [22,23]. Improvement of patient compliance is premised on meeting patient needs [17]. Thus, mHealth apps demand systematic and theory-based design to meet patients’ real needs for chronic disease management.

### Design Methods of mHealth Apps

The existing design methods of mHealth apps mainly include traditional information technology (IT) design [24-27], activity-centered design [28], user-centered design [29], participatory design [30], and goal-directed design (GDD) [31]. These different design approaches consider user needs in different ways. Table 1 summarizes the characteristics of these methods. Compared with other methods, GDD concentrates on user goals instead of on the tasks or activities that users must accomplish [32]. Focusing on user goals can directly reveal user needs; in contrast, tasks or activities are intermediate steps that help users to reach their goals [31]. Therefore, to improve patient compliance by meeting their needs, GDD is the most appropriate of the existing design methods.

**Table 1:** Summary of common design methods for mHealth apps.

Method	Requirement analysis	Driving force	Multidisciplinary collaboration	User engagement	Applicable scope
TID^a^	Based on technical documents written by developers	Technical document	No	Low	User needs are clear and well defined
ACD^b^	Based on activities users would perform with the app	User activity	Yes	High	Pay attention to user experience, and focus on what activities should be enabled by the app
UCD^c^	Based on observation of user behaviors by guiding them to complete a series of user tasks concerned with the app	User task	Yes	High	Pay attention to user experience, and focus on what tasks users should perform with the app
PD^d^	Based on user decisions by inviting them to participate in the design process	User decision	Yes	Very high	Users have rich experience in using mHealth apps and are familiar with the design process
GDD^e^	Based on user goals when using the app	User goal	Yes	High	Pay attention to user goals, and user needs remain to be clearly defined

^a^TID: traditional information technology design.

^b^ACD: activity-centered design.

^c^UCD: user-centered design.

^d^PD: participatory design.

^e^GDD: goal-directed design.

**Figure 1:**
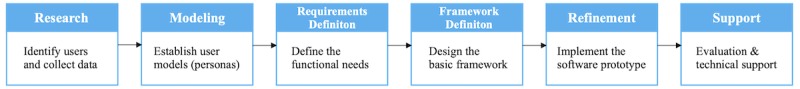
Workflow of the goal-directed design process.

The GDD design process can generally be divided into 6 phases: research, modeling, requirements definition, framework definition, refinement, and support. Figure 1 shows the workflow of the GDD design process. The research phase provides qualitative data about potential and/or actual users of the product through ethnographic field study techniques such as observation and contextual interviews. During the modeling phase, data discovered during the research phase are synthesized into user models. User models (personas) are detailed composite user archetypes that represent distinct groupings of behaviors, attitudes, aptitudes, goals, and motivations observed and identified during the research phase. For each primary persona, an analysis of the persona data and functional needs will be used during the requirements Definition phase. The output of this process is a requirements definition that provides a balance among different users. In the framework definition phase, software designers will create the overall product concept and define the basic frameworks for the product’s behavior and visual design. The output of this process is an interaction framework definition, which provides a logical and rough structure for the details to come. The refinement phase is similar to the framework definition phase but has an increased focus on details and implementation. Finally, the support phase offers support during and after development because even a very well-conceived and validated design solution cannot possibly anticipate every development challenge and technical question [31]. To summarize, GDD requires the modeling process before concrete design, and the user models, which are called personas, can capture user skills, environments, behaviors, and goals, etc. All these aspects of personas are intended to capture user needs [32].

### Objectives

Although GDD is prominent for identifying user needs and has gradually been adopted in practice, relatively little research concentrates on using GDD in the field of chronic disease management [33,34]. In fact, to the best of our knowledge, to date, few studies have used a theory-based design method to develop an mHealth app, particularly one intended to improve patient compliance with hypertension self-management. Therefore, to identify the needs of hypertensive patients and improve their compliance with hypertension self-management, we designed and implemented the Blood Pressure Assistant (BPA), an innovative monitoring and feedback tool that supports hypertension self-management and can be installed on patients’ smartphones. The GDD method was used to guide the design of BPA, and the end users (ie, hypertensive patients) were involved in the design process at an early stage. This paper describes the development process of BPA and the results of a pilot evaluation regarding patient compliance when using the BPA.

## Methods

### Study Design

**Figure 2:**
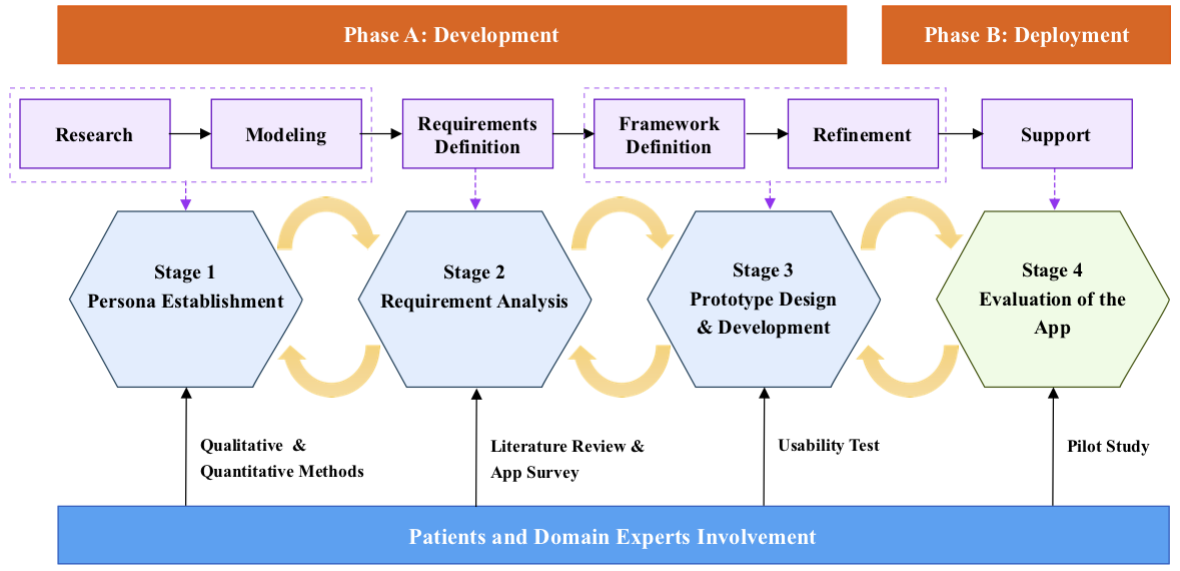
Complete study process.

This study was designed based on the GDD process. To simplify the design process and highlight the app evaluation, we made some modifications to the original GDD and divided the study into 4 iterative stages. Stages 1 to 3 covered the development phase, while stage 4 was the deployment phase. Figure 2 illustrates the complete study process and the comparison between the original GDD and our method. Stage 1 corresponds to the research and modeling phases, in which we used qualitative and quantitative methods to identify user personas. Stage 2 corresponds to the requirements definition phase, in which we defined the functional modules of BPA based on the identified user personas. Stage 3 corresponds to the framework definition and refinement phases, in which we designed the prototype of BPA based on the functional design and implemented it. Stage 4 corresponds to the support phase, in which we deployed BPA to the production environment and tested its effectiveness. Throughout the study process, patients and several domain experts (ie, experienced physicians) were involved, and they helped design the app and evaluate its effectiveness. The details of each phase are explained in the following sections.

### Participant Selection and Sampling

This study was conducted at the General Hospital of Ningxia Medical University, Ningxia Province. Participants in this study were outpatients and inpatients from the department of cardiology. Several physicians helped recruit patients into the study. The inclusion criteria were as follows: (1) aged 18 years or older, (2) a hypertension diagnosis with no other serious complications, (3) owned a smartphone and had home internet access, and (4) able to read and write in Chinese. Patients who met all the inclusion criteria were considered qualified to participate in the study. A random sampling method was applied by the researchers to ensure that the participating patients represented diverse personas.

### Informed Consent and Ethical Consideration

After sampling, the researchers contacted the selected qualified patients, apprised them of the goal of the study, and promised that their personal information would be accessed only by the researchers or the domain experts. The patients who agreed to participate in the study were asked to sign informed consent forms. The domain experts signed informed consent forms as well. All procedures were performed in accordance with the ethical guidelines for biomedical research involving human subjects at Ningxia Medical University.

### Goal-Directed Design Steps

#### Specified User Modeling Process of Goal-Directed Design

In our study design based on GDD, the first step is to model the users to establish their personas. However, the original user modeling process of GDD is generalized and abstract, which makes it difficult to use to specifically guide the design of mHealth apps for chronic disease management. Therefore, before using GDD to guide the design of BPA, we first specified the user modeling process of GDD for chronic disease management based on the following 2 points: (1) extracting user model elements specifically targeted to improve patient compliance with self-management regimes and (2) defining a concrete procedure for user modeling.

The authors of GDD indicated that user models can be described by 3 types of factors: demographic characteristics, domain expertise, and technical expertise [31]. We used evidence-based methods to extract specific elements of these factors from relevant studies and theories. Demographic characteristics are the most fundamental ways to describe users and usually include information such as gender, age, and occupation. With the development of mHealth technology in chronic disease management, several demographic characteristics have been found to specifically affect the patient self-management compliance. LeRouge et al [34] found that changes in the physiological characteristics of elderly people such as vision, cognitive, memory, and learning ability led to additional requirements for the functional design of mHealth apps. Grindford et al [35] found educational attainment to be related to the acceptance of mHealth technology interventions. In addition, career status (if retired) and patient postdiagnosis have been shown to influence patient self-management experiences [36]. Therefore, we defined age, educational attainment, career status, and postdiagnosis as the specific elements of demographic characteristics.

Domain expertise refers to user proficiency in related fields. We summarized the domain expertise of chronic disease patients from a number of aspects. First, self-management reflects a patient’s behavior in promoting, protecting, or maintaining their own health, and such behaviors can be explained by the health belief model (HBM) [37]. The HBM suggests that people’s perceived health threats, perceived benefits of action and barriers to action, and their self-efficacy explain their engagement (or lack of it) in health promotion behavior [38]. In this study, a perceived health threat means patients’ awareness of and concern for their hypertensive condition and its potential consequences. The perceived benefits of action and barriers to action refer to patients’ awareness of the benefits of health behavior changes and their resistance to such changes. Self-efficacy reflects the extent of patients’ beliefs in their ability to complete various tasks and reach the goal of controlling their hypertensive condition. Second, the positive effects of doctor-patient interactions for chronic disease management have long been established [39]. The doctor-patient relationship is an important factor that affects patients’ acceptance of mHealth interventions [40]. We thus proposed perceived health threat, perceived benefits of action and barriers to action, self-efficacy, and doctor-patient relationship to be the specific elements of domain expertise.

In the mHealth field, technical expertise refers to user acceptance of and proficiency with mHealth technology. The technology acceptance model (TAM) [41] is a classic theoretical model that has been widely used to predict consumer acceptance of mHealth technology [42-44]. According to TAM, perceived usefulness and perceived ease of use are the two major cognitive determinants of health information technology use [45]. Perceived usefulness refers to the extent to which users believe that using a particular app would enhance their task performance. Perceived ease of use is the extent to which users believe that using a particular app would be easy [41]. Moreover, users’ prior experiences with using technology can shape their beliefs about the new technologies [46]. Positive experiences may help patients feel more confident that they have the capabilities and resources to repeat that same performance with new technology [45,46]. Therefore, we defined perceived usefulness, perceived ease of use, and prior technology use experience as the specific elements of technical expertise.

**Figure 3:**
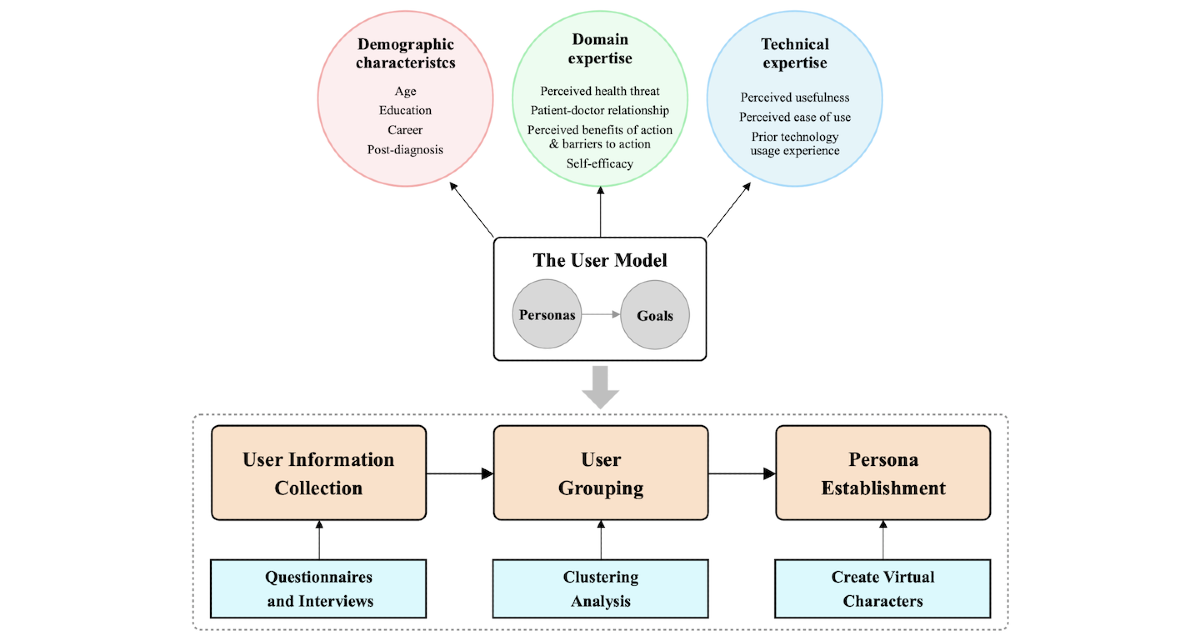
Specified user model for improving compliance with hypertension self-management and the concrete user modeling procedure.

After specifying the elements of the user model, we proposed the concrete user modeling procedure for our study based on the original GDD. The process for user modeling can be divided into 3 steps: (1) user information is collected through questionnaires and interviews based on the user model components, (2) statistic methods such as clustering analysis are used to group users and identify the personas and goals of each group, and (3) representative virtual characters (ie, persona) are created for each group describing their main characteristics and goals. Figure 3 shows the proposed user model and the concrete user modeling procedure for this study. Based on the specified user modeling process, we performed our 4-stage GDD method to guide the design of BPA.

#### Stage 1: Persona Establishment

After participant recruitment, we used questionnaires to collect patient information based on the user model, including demographic characteristics, domain expertise, and technical expertise (see Multimedia Appendix 1 for original questionnaires). The demographic characteristics questionnaire was in the form of fill-in-the-blank and included 5 items: gender, age, educational attainment, career status, and postdiagnosis. The domain expertise questionnaire consisted of 2 parts: the Awareness Rate of Hypertension Knowledge Scale (ARHKS) [47] and the Compliance of Hypertensive Patients Scale (CHPS) [48]. ARHKS contains 8 items in the form of multiple choice and has been widely used in relevant studies. CHPS contains 10 items extracted from the original version using a 4-point Likert scale. ARHKS was originally in Chinese. CHPS was translated into Chinese, and its reliability and validity have been examined [49]. We merged the scores of CHPS and ARHKS to obtain a total score for the domain expertise. The technical expertise questionnaire contained 10 items extracted from our previous study and used a 5-point Likert scale; it was mainly intended to evaluate patient acceptance of mHealth technology for hypertension self-management [50].

We then explored patient expectations of using the mHealth app through qualitative interviews. The interview included 4 open questions that had previously been discussed and validated by one researcher and one physician. The validated questions are as follows: (1) How do you manage your high blood pressure? Is it effective? In what ways can you do better? (2) In what ways do you hope to get help? (3) Are you willing to use your smartphone for self-management of hypertension? Why or why not? (4) What problems do you expect the app to help you solve? The researchers conducted the interviews in separate face-to-face sessions with each patient using a semistructured approach in which the researchers were freely able to change the order of the questions and could ask follow-up questions to ensure that the respondents’ answers were correctly understood. The interview was piloted on 3 patients to test content validity.

Using the questionnaire and interview results, we applied quantitative and qualitative methods to establish user personas. According to the specified user modeling process, a clustering analysis was first conducted to group the users. In this study, we chose the K-means algorithm and performed a clustering analysis on the domain expertise and technical expertise of the patients. Based on the grouping results, we then analyzed the differences between different groups regarding their demographic characteristics, domain expertise, technical expertise, and expectations of using the mHealth app. Finally, we created a virtual character (persona) for each group that reflected their characteristics and goals.

#### Stage 2: Requirement Analysis

In this stage, we defined what functional modules should be contained in BPA to meet patient needs and improve their compliance. We first investigated which functional modules have previously been included in existing mHealth apps from the literature and by examining real apps. We searched for literature on PubMed using a combination of terms relevant to mHealth technology for chronic disease management: “chronic disease,” “hypertension,” “mobile,” “smartphone,” “design,” and “development.” We searched for apps and downloaded them from the Tencent app store (one of the biggest app stores for Android apps in China) and the App Store (for iOS apps) using keywords “hypertension,” “diabetes,” “chronic disease,” and “health.” A total of 36 references and 37 apps were screened and analyzed (see Multimedia Appendix 2 for screened lists of references and apps). The concrete functional design was proposed corresponding to the established user personas. Different functional modules were designed to meet different patient needs, and design results were validated by the domain experts.

#### Stage 3: Prototype Design and Development

After identifying the functional modules of BPA, we designed the prototype and implemented it. First, we defined the main BPA interface, which organizes the concrete functional modules via the interface in different forms. We then designed a prototype of each functional module. We invited domain experts and patients to take part in the prototype development process. The experts evaluated the details of each functional module based on their knowledge and experience, and the patients were led by the researchers to join in the usability tests. After several iterations of the above 3 stages, we finally obtained a high-quality app prototype.

#### Stage 4: Prototype Evaluation

After BPA development, to further evaluate whether the app truly worked, we conducted a pilot study in which patients were provided with a copy of BPA installed on their own smartphones and were required to use it in their daily lives for 2 months. At the end of the trial period, we collected the patients’ uploaded data, calculated their compliance with their self-management regimes, and conducted interviews with them to identify the reasons for the compliance results and gather suggestions for further improvement of BPA.

### Data Analysis

Questionnaires were in the form of paper or online surveys. Paper questionnaires were collected and recorded into the database immediately after completion, while online questionnaires were automatically saved in the database. Disqualified questionnaires (with incomplete or contradictory answers) were discarded before the data analysis. All interviews were audiorecorded with the consent of the respondents, and all recordings were transcribed verbatim. The transcripts were generated, read, and open-coded using the NVivo 2.0 (QSR International) software package. Two researchers independently open-coded the transcripts. SPSS Statistics V20.0 (IBM Corp) and Python 3.0 were used for statistical analyses. Descriptive data is presented as counts and percentages. Multivariate analyses were conducted using Pearson chi-square tests for categorical variables and one-way analyses of variance for continuous variables. A *P* value of <.05 was considered statistically significance.

**Figure 4:**
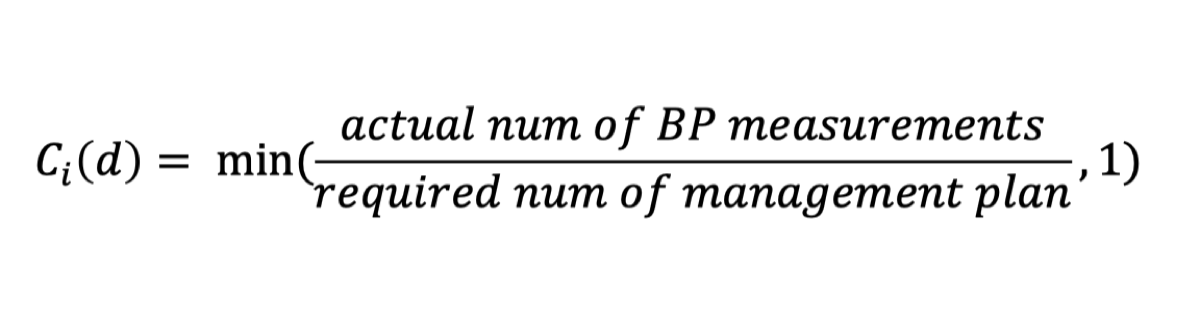
Compliance of one user on a specific day.

For the compliance analysis, in this study, patient compliance was defined as the ratio of actual frequency of BP measurements to the number required by the management plan. This metric was proposed by the researchers and validated by the physicians. Figure 4 shows the calculation process, in which *C_i_*(*d*) corresponds to the compliance of user *i* on a specific day *d*.

**Figure 5:**
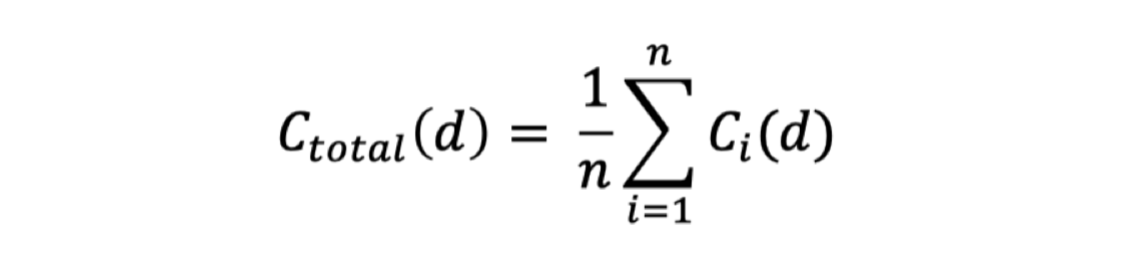
Average compliance of all groups for each version on a specific day.

In the evaluation stage, we used 2 specific formulas based on the above formula to evaluate patient compliance. The first formula calculated the average compliance of all patients for each day using the different app versions. As shown in Figure 5, *C_total_*(*d*) corresponds to the average compliance of all groups for each version on a specific day *d*, and *n* corresponds to the total numbers of all groups for each version.

**Figure 6:**
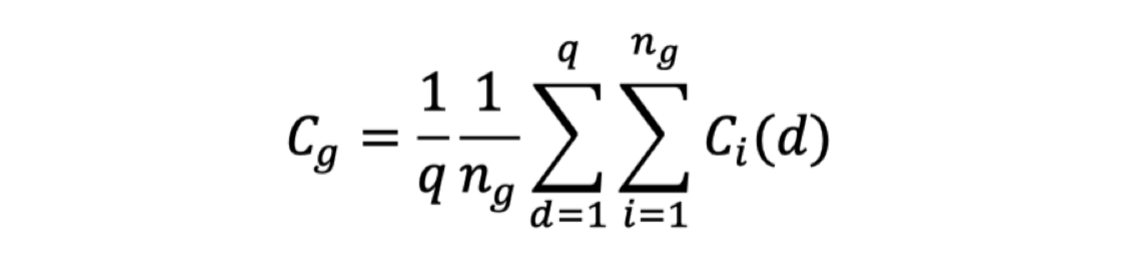
Average compliance of each group for each version.

The second formula calculated patient compliance during the entire trial for the different groups. As shown in Figure 6, *C_g_* corresponds to the average compliance of each group for each version, *n_g_* corresponds to the numbers of group *g* for each version, and *q* corresponds to the total days.

## Results

### Stage 1: Persona Establishment

**Figure 7:**
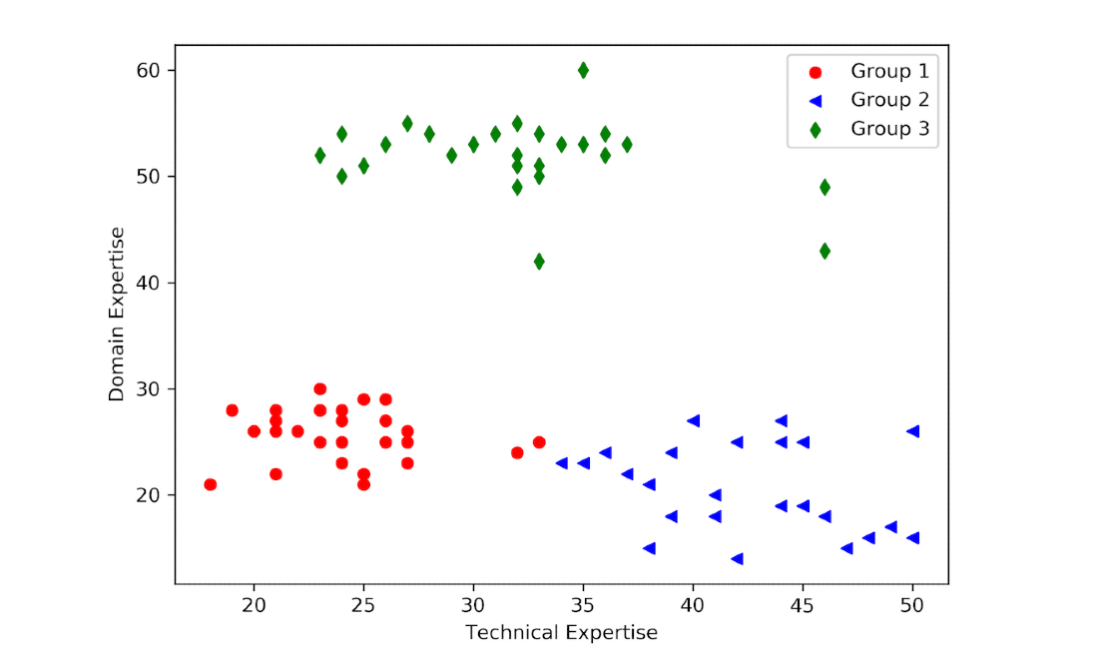
Clustering results on participant domain expertise and technical expertise.

A total of 90 questionnaires were distributed, and 82 valid questionnaires were collected. Answers to the domain expertise and technical expertise questionnaires were coded into points ranging from 0 to 50 and 0 to 60, respectively. The clustering results based on these 2 questionnaires are shown in Figure 7. These results demonstrated that hypertensive patients can be divided into 3 groups (the number of clusters was determined by the elbow method). A one-way analysis of variance showed that there were significant differences in domain and technical expertise among these 3 groups (*P*<.001). Based on the grouping results, we conducted statistical analyses on the demographic characteristics of different groups, and the results are shown in Table 2.

**Table 2:** Demographic characteristics of different groups.

Characteristics		Group 1 n=28	Group 2 n=26	Group 3 n=28	*P* value
**Sex, n (%)**					**.329**
	Female	13 (46)	7 (27)	10 (36)	
	Male	15 (54)	19 (73)	18 (64)	
Age in years, mean (SD)		53.8 (1.82)	49.7 (1.91)	62.3 (2.22)	<.001
**Career, n (%)**					**<.001**
	Employed	6 (21)	19 (73)	10 (36)	
	Self-employed/unemployed	15 (54)	7 (27)	3 (11)	
	Retired	7 (25)	0 (0)	15 (54)	
**Educational attainment, n (%)**					**.002**
	Secondary school and below	13 (46)	3 (12)	5 (18)	
	High school	8 (29)	7 (27)	15 (54)	
	Graduate and above	7 (25)	16 (62)	8 (29)	
**Postdiagnosis, n (%)**					**<.001**
	<1 year	17 (61)	3 (12)	0 (0)	
	1-5 years	11 (39)	16 (62)	5 (18)	
	>5 years	0	7 (27)	23 (82)	

A total of 18 patients were interviewed; the numbers of interviewed patients from groups 1 to 3 were 7, 5, and 6, respectively. We coded user expectations of the mHealth app based on these interview results (Table 3). The most frequently mentioned expectation was increased communication between doctors and patients. Concretely, the patients hoped to receive self-management guidance from doctors and expected timely intervention when abnormal conditions occurred. On the other hand, some patients hoped to increase their disease cognition levels through self-management. They hoped to acquire health knowledge about hypertension and a professional analysis of their self-monitoring data. Moreover, some patients considered the mHealth app to be a convenient way to conduct self-management and expected it to provide functions such as step counting, online registration, wearable device support, and so on. In addition, patients who lacked smartphone experience suggested that the app should have high usability—for example, its functions and operation should be as simple as possible.

**Table 3:** Interview coding results.

User expectations and explanation		Group 1 n=7	Group 2 n=5	Group 3 n=6
**Increasing communication between doctors and patients**				
	Doctors’ guidance of self-management	7	1	3
	Intervention of abnormal condition	1	2	4
**Increase disease cognition level**				
	Acquire knowledge about hypertension	3	2	4
	Professional analysis of their self-monitoring data	0	4	1
**A convenient way for self-management**				
	Step counting	0	3	2
	Timed reminder	5	3	0
	Online registration	0	3	0
	Wearable device support	0	3	1
**High usability**				
	Simple operation	7	1	3
	Simple function	4	1	2
	Auto-uploading	1	3	1
	Increasing fun	0	3	0

Based on the above results, we established personas for hypertension patients in the different groups (Table 4). The personas reflected the differences between hypertensive patients at the demographic, domain expertise, and technical expertise levels. The needs (goals) of these 3 groups of patients for using hypertension self-management apps can be summarized as follows: to improve their self-management ability, enhance their self-management motivation, and receive self-management support.

**Table 4:** Personas of hypertensive patients.

Characteristics and goals	Group 1	Group 2	Group 3
Age in years	50	45	65
Career	Self-employed/unemployed	Employed	Retired
Educational attainment	Secondary school and below	Graduate and above	High school
Postdiagnosis	<1 year	1-5 years	>5 years
Experience in using smartphone	Low	High	Medium
Perceived ease of use of smartphone	Low	High	Medium
Disease cognition level	Low	Medium	High
Self-management ability	None	Poor	Good
Expectations to use hypertension self-management apps	Self-management under the doctors’ guidance; receive reminder of self-management; easy to use	Learn the disease progress; more fun in self-management; upload data automatically	Learn more knowledge related to hypertension; receive warning and intervention according to self-monitoring data
Goals	Improve self-management ability	Enhance self-management motivation	Receive self-management support

The patients in group 1 had a low level of disease cognition and lack of self-management experience. They wanted to manage their disease under the guidance of doctors and receive timely reminders and interventions. In addition, they demanded that the app must be easy to use because they lacked experience in using smartphones. Therefore, doctor supervision and an app with enhanced usability were the primary solutions to improving compliance of group 1.

The patients in group 2 were relatively young and better educated. They generally were more accepting of smartphone technology and had more experience in self-management, but they did not take their hypertensive condition seriously, and their compliance with medical orders was low. We considered providing self-management motivation to be the way to improve the compliance of group 2.

The patients in group 3 had a higher level of disease cognition and were capable of performing effective self-management at home. They wanted to learn more about their hypertension and receive feedback based on their self-monitoring data. Thus, further strengthening their self-management abilities and providing effective medical service support under abnormal conditions were the keys to improving compliance of group 3.

### Stage 2: Requirements Analysis

The results of investigating functional modules in existing mHealth apps are shown in Table 5. These results demonstrated that a variety of functional modules designed in existing mHealth apps focus primarily on self-monitoring. Only some of the apps considered supervision and intervention from doctors. Based on the investigation results and established user personas, we proposed specific solutions to meet the needs of patients in different groups. For patients with the desire to improve their self-management ability, concrete and executable plans with reminder services as well as doctor supervision were essential. For patients with the desire to improve their self-management motivation, we aimed to inform them about their disease control situation and used some gamification designs to enhance their motivation. For patients with the desire to receive self-management support, providing health education and timely intervention under abnormal conditions were important.

Different functional modules were designed based on the proposed solutions. For patients who aimed to improve self-management ability, the management plan module was designed to provide guidance to patients on how to conduct self-management at home. Management plans were formulated by doctors through a telehealth system, and the system automatically split plans into daily tasks and sent them to the patients’ smartphones. The management plan generally included BP and weight monitoring frequency, medication guidance, and advice on diets and exercise. Patients needed to record their health data according to the tasks, and the data would be sent to the doctors. The doctors needed to perform regular follow-ups with patients and adjust their management plans according to the uploaded health data. When the plans were changed, patients received new daily tasks on their smartphones instantly. In addition, a reminder service module was designed in order to prevent patients from forgetting to complete the tasks.

**Table 5:** Investigation results of functional modules in existing mHealth apps.

Category and functional module			References n=36		Apps n=37
Early detection: risk assessment			5		16
Disease cognition: health education			13		23
**Lifestyle intervention**					
	Recipes			1	11
	Exercise plan			2	3
**Disease management and control**					
	**Self-monitoring**				
		Blood pressure/blood glucose		36	37
		Weight		27	21
		Medication		12	35
		Diet		8	14
		Exercise		8	12
	Statistical report			11	20
	Reminder service			5	18
	Abnormal warning			8	4
**Intervention of doctors**					
	Short message service			12	7
	Telephone follow-up			6	4
	Online consultation			10	8
**Gamification design**					
	Challenge and reward			4	0
	Leaderboard			3	0
	Social contact			5	10

For patients who aimed to enhance their self-management motivation, we designed the health report and leaderboard modules. The health report module provided daily and monthly reports to patients according to their health data. The leaderboard module aimed to provide motivation for self-management. All the behaviors of patients concerned with self-management were converted into scores, and patients could view their real-time leaderboards to compare themselves with other users though the app (with no identifiable data are used).

**Figure 8:**
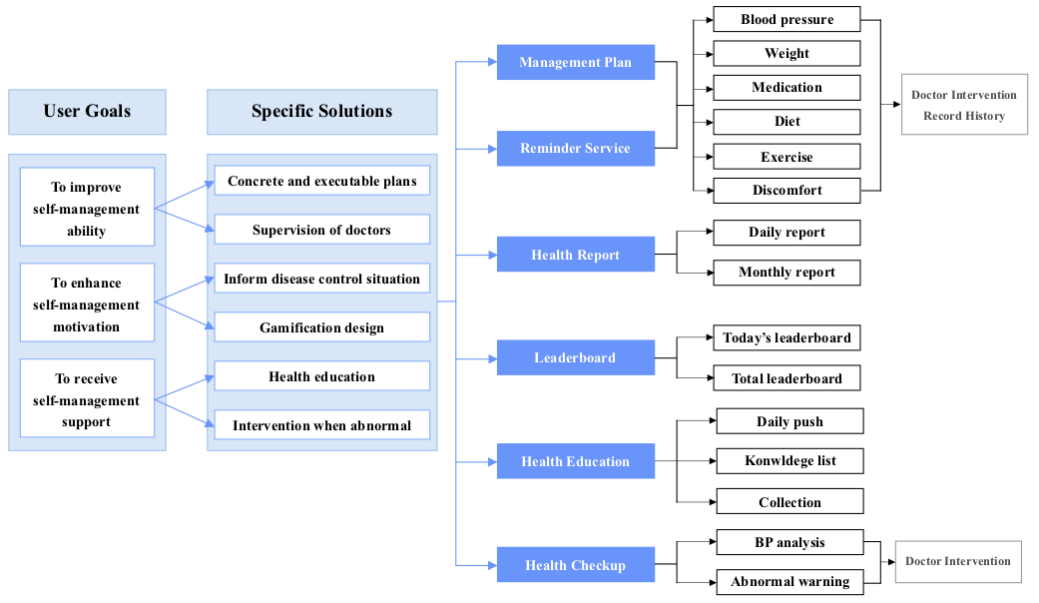
Complete requirements analysis process.

For patients who aimed to receive self-management support, the health education module provided health knowledge about chronic diseases to patients in various forms, and the health checkup module automatically analyzed the patients’ recorded B*P* values and sent feedback to the patients. When a patient’s B*P* value was abnormal, the doctors would be informed immediately through the system. The entire requirements analysis process is shown in Figure 8.

### Stage 3: Prototype Design and Development

#### Main Interface

**Figure 9:**
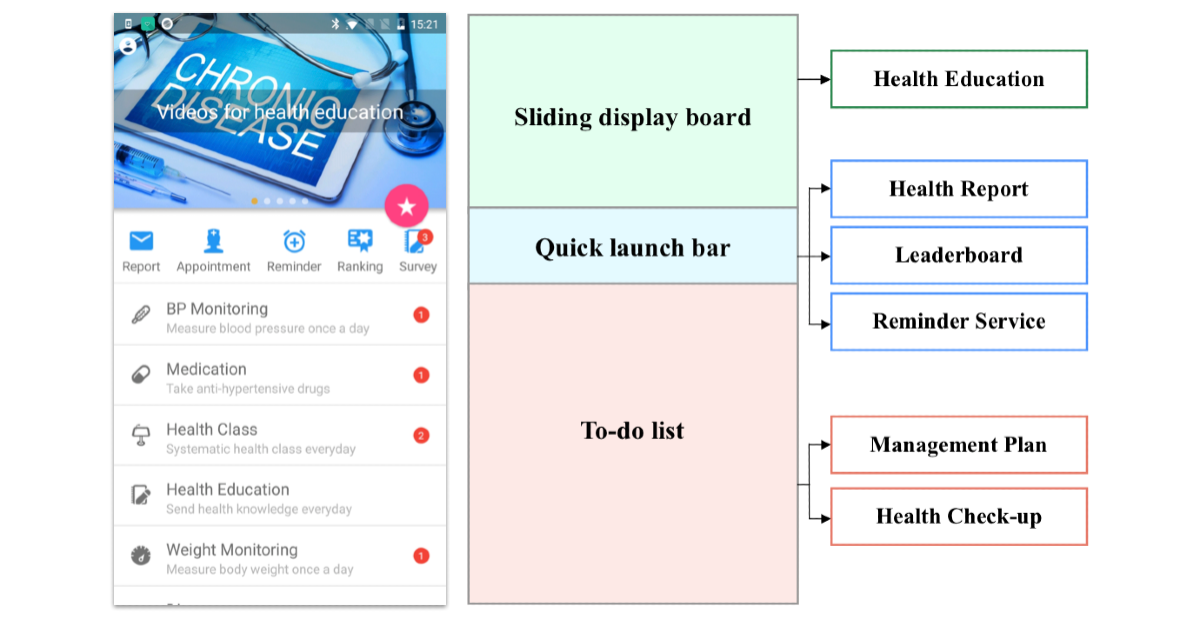
Main Blood Pressure Assistant interface.

The resulting design for the main interface is shown in Figure 9. We divided the main interface into 3 parts: to-do list, sliding display, and quick launch bar. The to-do list mainly shows a list of tasks in the management plan, along with a brief introduction to each task. Users could clearly see the completion status of all the tasks via the list, which included a red highlight next to uncompleted tasks. By clicking on a specific row, users could enter the corresponding task interface of the management plan. The sliding display board shows daily updated health knowledge. Health knowledge shown by sliding the display effectively attracted users’ attention while also serving as a quick entry point for reading the knowledge. Users could access the independent health education module to read knowledge as well (from the to-do list). The quick launch bar consisted of several small icons corresponding to different functional modules. Users could access the functional modules quickly by clicking on the icons. This part of the app was designed for less frequently used functional modules such as the leaderboard, health report, and reminder service.

#### Management Plan Module

The management plan module appeared as a to-do list in the main interface of BPA. The list of daily tasks generated from the management plans formulated by doctors was grouped into 5 items: recording health data such as BP and weight, recording medication, recording diet, recording exercise, and recording uncomfortable symptoms. Each task was required to be completed at a specific time during each day. When users clicked on an item, a series of to-do cards for the current task would appear in a new interface. Patients only needed to input the required data and click on an OK button on the card to finish a task. After completing a task, the color and shape of the card would change, indicating the task’s state transition. The input data were uploaded to the system database instantly so that doctors were able to remotely check up-to-date patient health records.

#### Reminder Service Module

The reminder service module was accessible from the quick launch bar or from within the management plan module. The reminder time was initially set based on the management plan, and when a time period elapsed, a dialog with the specific task would appear on the patients’ screens, along with an audible alarm. Users who were unable to perform the tasks according to the default schedule could set a reminder, forming a customized schedule for their daily self-management tasks. In addition, users were free to reset or close the reminders.

#### Health Report Module

The health report module was accessible through the quick launch bar. Users received daily reports and a monthly report consisting of 3 parts: user’s current health condition based on their historical data, uncompleted tasks, and the current day’s (or current month’s) score. Scores were calculated based on the completion status of provided tasks and on changes in health data. This functional module served to remind patients to accomplish any remaining tasks and motivated them to perform self-management tasks to achieve higher scores.

#### Leaderboard Module

Based on the scores obtained in the health report module, the leaderboard module provided competitive motivation for users by allowing them to compare their scores with those of other users. The module consisted of 3 parts: user’s score, leaderboard among all users, and current user’s rank among all users. In the daily leaderboard, users could view the top 30 users for the current day, while in the all-time leaderboard, users could view the top 30 users of all time. The design of leaderboard module applied a competitive strategy through persuasive technology [51] to motivate users to stick to their self-management regimes and strive for higher scores so their names would appear on leaderboards.

#### Health Education Module

Health education can improve disease awareness and help patients perform self-management more effectively. BPA provided 2 kinds of education. Educational articles and videos of common chronic diseases collected from the internet and vetted by the physicians covered all aspects of daily chronic disease management. Systematic educational material concerning hypertension based on guidelines and books was presented; this content was more professional and suitable for patients who wanted to learn more about their disease.

#### Health Check-Up Module

**Figure 10:**
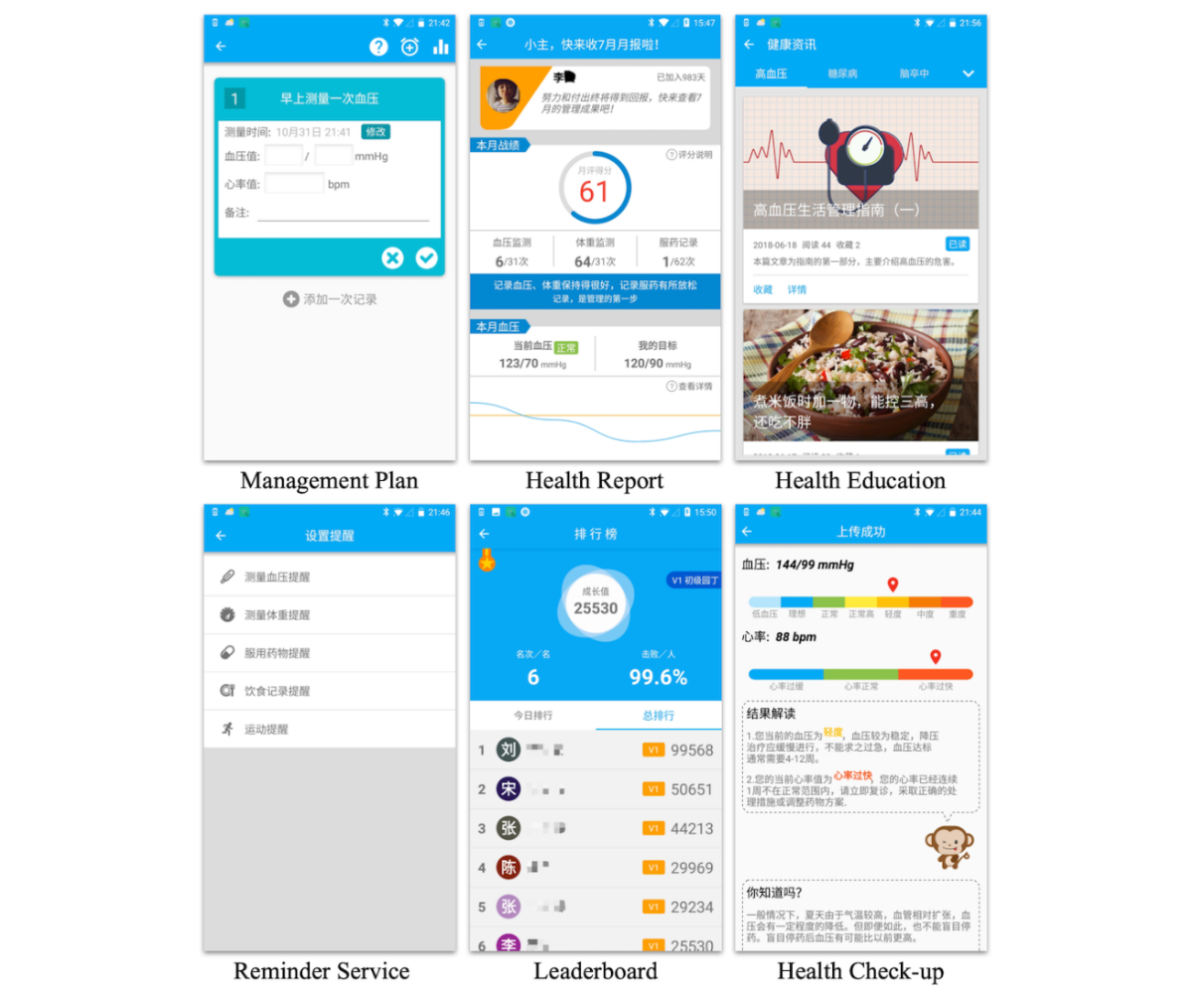
Screenshots of the 6 functional modules in the Blood Pressure Assistant.

Patients’ B*P* values are important indicators of their health condition. Each time a patient uploaded a B*P* value, the health check-up module would analyze that value by considering the patient’s BP trends and their hypertension grade and return feedback to the patient. If the B*P* value was considered to be abnormal, a warning message would be sent to the patient, asking them to take a rest and measure their BP again in 20 minutes. All abnormal conditions were also sent to doctors immediately through the telehealth system. Doctors should pay close attention to abnormal patients and conduct interventions when appropriate. Figure 10 shows screenshots of the 6 functional modules in BPA (see Multimedia Appendix 3 for detailed screenshots).

### Stage 4: Evaluation

#### App Version and Patient Grouping

**Figure 11:**
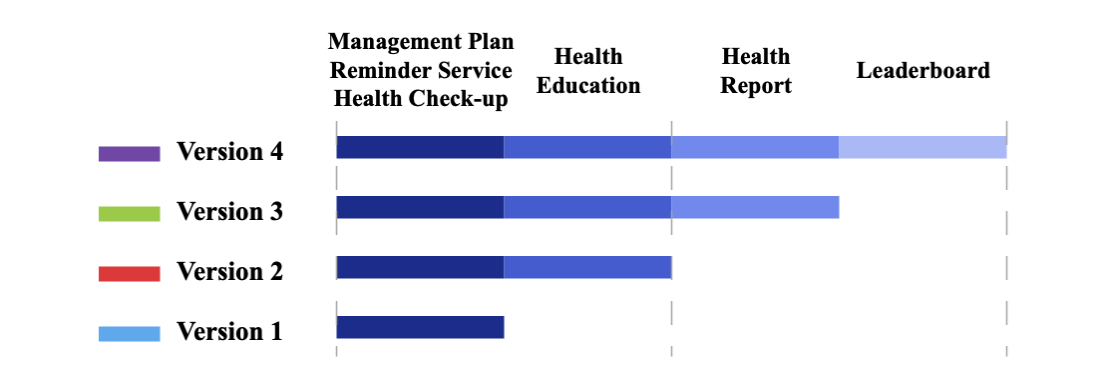
Functional modules contained in each version.

The development of BPA was an iterative process. We gradually recruited subjects as different functional modules were added to the app. Four versions of the app were released during the trial. Version 1 contained 3 main functional modules: management plan, reminder service, and health check-up. The other 3 modules were added in subsequent versions (Figure 11). Patients were required to use the different versions of the app for at least 2 months. A total of 143 patients completed the trial. We grouped all subjects according to the personas proposed in stage 1, and results are shown in Table 6.

**Table 6:** Numbers of patients in the different groups.

Group	Version 1 n=36	Version 2 n=39	Version 3 n=36	Version 4 n=32
Group 1	11	14	14	11
Group 2	14	10	12	9
Group 3	11	15	10	12

#### Compliance Analysis

**Figure 12:**
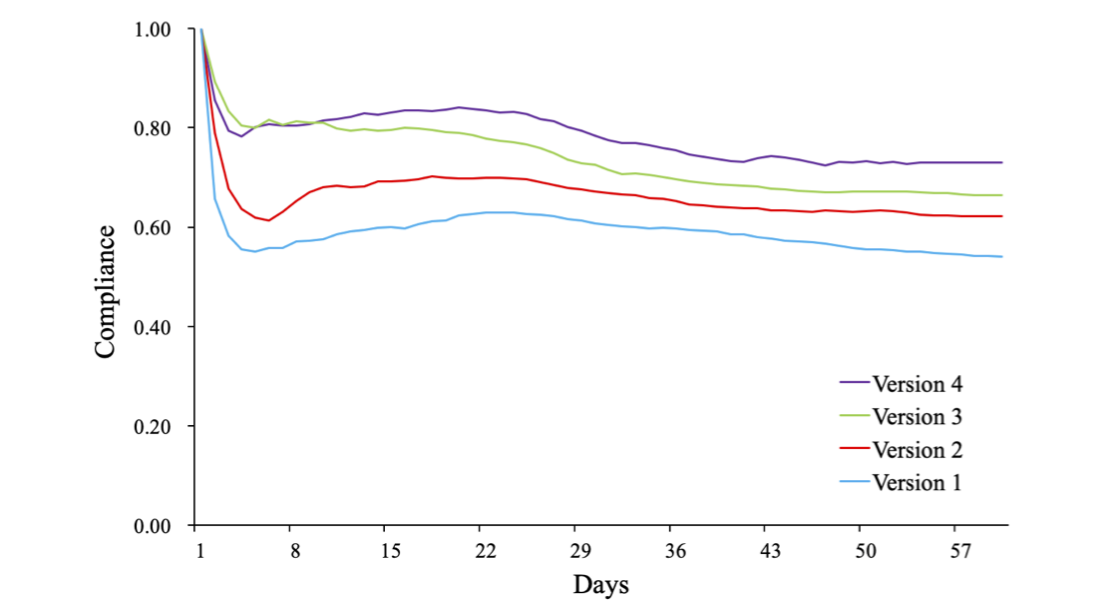
Compliance trends for each app over 2 months.

As mentioned in the data analysis section, we calculated the average compliance of all patients for each day using the different app versions. Each version was used for 2 months. Figure 12 shows the compliance trends of each version. The initial value of compliance was set to 1 on day 1. We can see that for all 4 versions, compliance first declined sharply and then gradually increased during the first week. The initial decline reflects the fact that patients were not familiar with the app and had not built trust in the doctors. The inflection point appeared on the third to the fifth day after becoming involved in the assisted self-management (ie, after starting to use the app), consistent with the time when the doctors conducted the telephone follow-up. This result indicates that timely doctor intervention can effectively alleviate user resistance during the initial stage when they receive new technology, thus improving their compliance. Compliance reached its highest level during the third week (except for the initial week) and slowly decreased to a stable level, indicating that time is another factor that affects patient compliance.

We calculated patient compliance during the trial for the different groups. Results show that patient compliance improved as functional modules were added to the app and was maintained at a high level (Table 7). Total compliance increased from 0.54 to 0.73, a significant difference (*P*<.001).

**Table 7:** Patient compliance among the different groups.

Group	Patient compliance					Analysis of variance	
	Version 1	Version 2	Version 3	Version 4	*F-*value		*P* value
Group 1	0.53	0.56	0.67	0.73	24.28		<.001
Group 2	0.39	0.51	0.50	0.59	14.64		<.001
Group 3	0.69	0.73	0.83	0.86	19.54		<.001
Average	0.54	0.62	0.66	0.73	11.30		<.001

To determine the reasons for patient compliance, we interviewed 32 patients from different groups who had used version 4 of the app. The interview included reasons for high or low compliance and suggestions for improving the app. Table 8 shows the coding results for the reasons mentioned in the interviews.

**Table 8:** Reasons for patient compliance from the interview.

Reason given		Group 1 n=11	Group 2 n=9	Group 3 n=12
**Reasons for high compliance**				
	Received doctors’ guidance	9	5	10
	Reminders and supervision	7	7	3
	Learned to control blood pressure	1	8	3
	Enriched related knowledge	7	4	7
	Enhanced pleasure	2	5	3
	Easy to use	5	0	2
**Reasons for low compliance**				
	Boring to record data	0	4	0
	Did not know how to use the app	4	0	0
	Did not have time	0	4	0

#### Reasons for High Compliance

Receiving doctors’ guidance and enriching related knowledge were the most frequently mentioned reasons. The doctors’ advice included the following: (1) for patients who had little experience in hypertension self-management, doctors taught the patients how to self-manage their disease and (2) for patients who had sufficient experience in hypertension self-management, doctors conducted timely interventions to help patients deal with abnormal conditions. Therefore, doctors played an important role in patient hypertension self-management, which can effectively improve patient compliance. In addition, health education can raise the self-management awareness of different groups, thus improving their compliance.

A total of 17 patients (mainly in groups 1 and 2) mentioned that the BPA app played a role by providing reminders and supervision. For patients who lacked experience in self-management, the reminder service in BPA reminded them to complete the daily tasks, and for patients who lacked motivation in self-management, regular follow-ups from doctors encouraged them to pay more attention to their self-management.

Twelve patients (mainly in group 2) agreed that BPA helped patients learn how to control their hypertension. Patients in group 2 were more highly educated and interested in learning more about their disease. The health report module was helpful for improving their compliance.

Ten patients mentioned that the app helped enhance the pleasure of self-management. The leaderboard module provided social support for patients and improved their compliance by indirectly improving their motivation. Seven patients (mainly in group 1) mentioned ease of use, which is considered particularly important for patients who lack smartphone experience.

#### Reasons for Low Compliance

Four users in group 2 mentioned that it was boring to record data. We considered integrating wearable devices with the app to simplify the recording process. However, the main reason for noncompliance is that patients have no intrinsic motivation to monitor their health indicators. Therefore, the solution to this problem is to further increase patient motivation.

Some patients did not know how to use the app. This aspect was mentioned by 4 users in group 1. Patients in this group had relatively low educational levels and often lacked experience using smartphones. Teaching them how to use the app during the initial period of management is the key to solving this problem.

Some patients said that they had no time to manage their disease. Four users in group 2 mentioned this problem. However, we think that an additional important reason is a lack of intrinsic motivation. Similar to the first reason, the path toward changing users’ behaviors is to add extrinsic motivations that make them pay more attention to their disease management.

#### Suggestions for Further Improvement

Five patients suggested increasing communication between doctors and patients. The current BPA adopted a one-way form of communication—from doctors. However, patients wanted to be able to take the initiative to communicate with their doctors. Three patients suggested an online registration function to save time and improve treatment efficiency. In the current version of BPA, we have added this module, but its usability remains to be tested. Six patients hoped to automatically measure BP data using their smartphones instead of having to manually input the data after measuring their BP through wearable devices. This requirement stemmed from the fact that these patients often traveled for business, which made it difficult for them to monitor their BP regularly. Being able to use their smartphones to measure their BP might increase their self-monitoring frequency. However, due to the technological limitations, achieving accurate BP measurements using unmodified smartphones remains a future goal [52].

## Discussion

### Principal Findings

In this study, we designed an innovative mHealth app (BPA) for hypertension self-management and evaluated patient compliance with its use. To ensure that the app met patients’ needs, we used the GDD method and involved patients in every stage of the design process. Three major patient goals (needs) were identified: improve self-management ability, enhance self-management motivation, and receive self-management support. Different functional modules of BPA were designed based on the identified patient needs. To evaluate the effectiveness of the BPA app, we invited 143 patients to use different versions of BPA for 2 months. Patient compliance with BPA version 4 reached 73%, a favorable result. According to interviews, patients felt that BPA taught them how to take the right actions to manage their disease, and they felt encouraged when instructed by doctors. Results suggest that patients are willing to try new tools with new technologies if those tools are designed specifically for them. We also identified some problems existing in patient self-management of hypertension. Some of the reasons for low compliance were attributable to low motivation for disease management. Patients should be provided with additional extrinsic motivation in multiple ways. Another possible improvement for BPA was that it should help build a closer relationship between patients and doctors. A communication module could be added to the app to fulfill this need.

### Comparison With Prior Work

To the best of our knowledge, this study is the first to explicitly use GDD principles to develop an mHealth app for hypertension self-management. To understand the innovation of our study, we compared it with 5 prior studies that used a theory-based design method in the chronic disease management field. The results are shown in Table 9. Among these studies, two (Fore et al [33] and our study) explicitly used GDD as the design method. LeRouge et al [34] investigated user-centered design as a methodological tool but also established user personas during the design process. Five of the studies ([34,53-55] and ours) designed smartphone apps for specific diseases. Two studies (Morita et al [55] and ours) provided evaluations of their design results. In addition, Wachtler et al [54] reported that their tool was being evaluated in a randomized controlled trial.

**Table 9:** Comparison of recent studies using theory-based design for disease management.

Study	Country	Design method	Objective	Disease	Sample size for design	Final output	Evaluation
Our study	China	GDD^a^	Improve patient compliance with hypertension self-management	Hypertension	90 questionnaires and 18 interviews	Smartphone app	Yes
Fore et al [33]	United States	GDD	Improve chronic illness care	Pediatric inflammatory bowel disease	10 patients, 10 caregivers, 10 physicians, and 6 nurses (interviews and observations)	Prototype of a learning health system	No
LeRouge et al [34]	China	UCD^b^	Identify user profiles and personas of an aging population	Diabetes	9 focus groups, interviews with 21 physicians and 9 nurses	Smartphone app	No
van der Weegen et al [53]	Netherlands	UCD	Stimulate physical activity of people with a chronic disease	Chronic obstructive pulmonary disease or type 2 diabetes	15 interviews with patients, 2 focus groups, 16 interviews with health care professionals, and discussion with several experts	Triaxial activity sensor along with smartphone app	No
Wachtler et al [54]	Australia	UCD	Improve treatment allocation for depression	Depression	2 focus groups with community sample (n=17) and 7 interviews with people with depressive symptoms	Smartphone app	No
Morita et al [55]	Canada	UCD	Support asthma self-management	Asthma	11 interviews and 5 usability tests	Web-based mHealth platform	Yes

^a^GDD: goal-directed design.

^b^UCD: user-centered design.

Compared with these prior works, our study was innovative in a few ways. User models (personas) were established based on elements concerned with patient compliance rather than simply considering all factors. Elements were extracted mainly from relevant theories such as HBM and TAM.

In the persona establishment stage, qualitative and quantitative methods were used together to identify user personas. Qualitative methods are appropriate for dividing users into specific groups, while quantitative methods are suitable for extracting the intrinsic needs of different groups.

Different functional modules were designed for different personas and integrated into a complete mHealth app by following a reasonable priority. For example, the management plan module appears in the most conspicuous position of the main interface because it is the most frequently accessed part. In contrast, the leaderboard module is accessible through the quick launch bar of the main interface because it is less frequently used and is designed primarily for experienced users.

### Strengths

Our study has several strengths. First, it verified the viewpoint that patients should self-manage their disease under the direction of doctors [56]. Only in this way can patients conduct comprehensive self-management and have abnormal conditions handled in a timely manner. This approach also accounted for the reasons why BPA was more effective compared with other apps available in app stores.

Second, we involved experts and patients in the design process. Chronic disease patients consist primarily of elderly people who have special and additional disease self-management needs [34]. To better understand the needs of these patients, we used questionnaires and interviews to identify the different personas of different patient groups. These personas were used to guide the functional design. In addition, elderly people are less confident in the use of mobile technologies. As a result, more attention should be paid to the usability of such apps, which is an important factor affecting patient compliance. Inviting users to participate in the design process can help discover usability problems in a very early stage.

Third, we proposed a new compliance assessment method in which the self-monitoring frequency of BP was considered as an evaluation index for hypertension self-management compliance. The most common approach for measuring patient compliance has previously been to ask patients for their ratings of compliance behavior or acquire the information through standardized questionnaires [10]. However, such approaches may result in higher compliance levels than the true levels due to patient subjectivity and memory bias [57]. Compliance with medication regimes is the most evaluated index according to several studies [58-60] because that is the most direct indicator of compliance with the doctor’s instructions. However, taking medicine on time is easier to perform than other self-management tasks [61]; consequently, such a metric cannot fully reflect the complete content of patients’ self-management. Compared with other studies, we used the frequency with which patients self-monitor their B*P* values and collected the results automatically through the app. Thus, this evaluation result was more objective and practical over the long term. Although our study was only a preliminary trial, it helps set a benchmark that further research can refer to when assessing such apps.

### Limitations and Future Works

One limitation of our study is that the number of recruited subjects for persona establishment was relatively small, and most were highly educated. Therefore, the generalizability of the study’s results needs to be further confirmed. Another limitation is that the pilot study lasted for only 2 months; patient compliance over a longer period of time is unknown. The interview results show that a few patients with low compliance still exist, mainly due to a lack of intrinsic motivation. In addition, one other thing to note is that in eHealth enhanced chronic disease management, patient compliance is influenced by multiple factors such as well-designed self-management tools, community-based eHealth support, and a complete feedback loop between patients and doctors [62]. Our study focused on the functional design of self-management tools, which is one of the important factors influencing patient compliance.

In future work, we will include more patients and test for a longer time span. Comparison tests should be conducted to determine how much patient compliance is improved. We also plan to compare patient compliance as measured by this study’s approach with compliance measured by a traditional approach, such as questionnaires, to further explore the practical validity of our approach. In addition, we need to find a way to further increase patient motivation for self-management.

### Conclusions

The BPA app was shown to be an effective tool for hypertension self-management in terms of patient compliance. The GDD method was able to identify the different needs of different patients and guided functional design based on these needs. We learned from the study that efficient self-management of hypertension should be led by doctors. Usability is another important factor that should be considered during the development of apps. In addition, hypertensive patients should receive more extrinsic motivation in the management of their disease.

## Appendix

Multimedia Appendix 1 Original questionnaires.

Multimedia Appendix 2 Screened lists of articles and apps in stage 2.

Multimedia Appendix 3 Detailed screenshots in the Blood Pressure Assistant.

## Abbreviations

ARHKS: Awareness Rate of Hypertension Knowledge Scale
BP: blood pressure
BPA: Blood Pressure Assistant
CHPS: Compliance of Hypertensive Patients Scale
GDD: goal-directed design
HBM: health belief model
IT: information technology
mHealth: mobile health
TAM: technology acceptance model
