# Correction: Using Goal-Directed Design to Create a Mobile Health App to Improve Patient Compliance With Hypertension Self-Management: Development and Deployment

**DOI:** 10.2196/18859

**Published:** 2020-05-28

**Authors:** Huilong Duan, Zheyu Wang, Yumeng Ji, Li Ma, Fang Liu, Mingwei Chi, Ning Deng, Jiye An

**Affiliations:** 1 College of Biomedical Engineering and Instrument Science Ministry of Education Key Laboratory of Biomedical Engineering Zhejiang University Hangzhou China; 2 General Hospital of Ningxia Medical University Yinchuan China

In “Using Goal-Directed Design to Create a Mobile Health App to Improve Patient Compliance With Hypertension Self-Management: Development and Deployment” (JMIR Mhealth Uhealth 2020;8(2):e14466), there was an error which was not identified during the proofreading stage.

The first five sentences and the last word of the Methods subsection in the Abstract were missing in the published paper. The original published Methods subsection of the Abstract was incorrectly presented as:

Clustering methods based on questionnaire responses were used to group patients. Qualitative interviews were conducted to identify the needs of different groups. In stage 2, several functional modules were designed to meet the needs of different groups based on the results from stage 1. In stage 3, prototypes of functional modules were designed and implemented as a real app. Stage 4 was the deployment process, in which we conducted a pilot study to investigate patient compliance after using the app. Patient compliance was calculated through the frequency with which they took blood pressure measurements. In addition, qualitative interviews were conducted to learn the underlying reasons for the compliance

The correct Methods subsection of the Abstract is:

The goal-directed design method was applied to guide study design. We divided the study into 4 stages. Stages 1 to 3 comprised the development process. To improve the applicability of the goal-directed design method to chronic disease management, we extracted elements of user models concerned with patient compliance and defined a concrete process for user modeling. In stage 1, personas of hypertensive patients were built using qualitative and quantitative methods. Clustering methods based on questionnaire responses were used to group patients. Qualitative interviews were conducted to identify the needs of different groups. In stage 2, several functional modules were designed to meet the needs of different groups based on the results from stage 1. In stage 3, prototypes of functional modules were designed and implemented as a real app. Stage 4 was the deployment process, in which we conducted a pilot study to investigate patient compliance after using the app. Patient compliance was calculated through the frequency with which they took blood pressure measurements. In addition, qualitative interviews were conducted to learn the underlying reasons for the compliance results.

The changes made do not affect the findings of the study.

In addition, the original published paper contained an error in author affiliation 1. Affiliation 1 was incorrectly listed as:

College of Biomedical Engineering and Instrument Science, Ministry of Education Key Laboratory of Biomedical Engineering, Hangzhou, China

The correct listing for author affiliation 1 is:

College of Biomedical Engineering and Instrument Science, Ministry of Education Key Laboratory of Biomedical Engineering, Zhejiang University, Hangzhou, China

The correction will appear in the online version of the paper on the JMIR website on May 28, together with the publication of this correction notice. Because this was made after submission to PubMed, PubMed Central, and other full-text repositories, the corrected article has also been resubmitted to those repositories.

