# Supplementary material for: Using Goal-Directed Design to Create a Mobile Health App to Improve Patient Compliance With Hypertension Self-Management: Development and Deployment
Source: JMIR Mhealth Uhealth. 2020 Feb 25;8(2):e14466. doi: 10.2196/14466 (PMC7064970; doi:10.2196/14466)
Supplement: Multimedia Appendix 1 [file mhealth_v8i2e14466_app1.docx]

**Questionnaire 1:** **Awareness Rate of Hypertension Knowledge scale**

1. What’s the diagnostic criteria for hypertension?
   1. 150/100 mmHg
   2. 140/90 mmHg
   3. 130/80 mmHg
   4. 130/90 mmHg
   5. I don’t know
2. Is hypertension a lifelong disease?
   1. Yes, it’s incurable but can be controlled
   2. No, it can be cured
   3. I don’t know
3. Which of the following factors do you think are related to hypertension? (multiple choice)
   1. Overweight or obesity
   2. Heredity
   3. Smoking and drinking
   4. Too much salt intake
   5. I don’t know
4. If hypertension is not well controlled, which of the following diseases will it result in? (multiple answers)
   1. Coronary heart disease
   2. Stroke
   3. Renal insufficiency
   4. Hypertension will not result in any other disease
   5. I don’t know
5. How to prevent hypertension? (multiple answers)
   1. Low-salt diet
   2. Exercise
   3. Control body weight
   4. Smoking cessation
   5. Quit alcohol
   6. Keep psychological balance
   7. I don’t know
6. What’s the daily salt intake recommendation?
   1. 10 g
   2. 8 g
   3. 6 g
   4. 4 g
   5. I don’t know
7. Do you think a hypertensive patient should keep taking medicine when his/her blood pressure is well controlled?
   1. Yes, he/she must insist on medication
   2. No, he/she only need to take medicine when the blood pressure is not well controlled
   3. I don’t know
8. Do you think the blood pressure of a hypertensive patient should fall as low as possible?
   1. Yes, of cause as low as possible
   2. No, it depends on age and degree of organ damage
   3. I don’t know

**Questionnaire 2: Compliance of Hypertensive Patients scale**

1. For hypertension self-management, I have a clear goal
   1. Strongly agree
   2. Agree
   3. Disagree
   4. Strongly disagree
2. I am willing to develop and execute the hypertension self-management plan along with doctors
   1. Strongly agree
   2. Agree
   3. Disagree
   4. Strongly disagree
3. I have a positive attitude toward hypertension treatment
   1. Strongly agree
   2. Agree
   3. Disagree
   4. Strongly disagree
4. I am willing to change unhealthy lifestyle
   1. Strongly agree
   2. Agree
   3. Disagree
   4. Strongly disagree
5. I am willing to obey low fat diet
   1. Strongly agree
   2. Agree
   3. Disagree
   4. Strongly disagree
6. I am willing to obey low salt diet
   1. Strongly agree
   2. Agree
   3. Disagree
   4. Strongly disagree
7. I am willing to take exercise
   1. Strongly agree
   2. Agree
   3. Disagree
   4. Strongly disagree
8. I am willing to monitor my blood pressure according doctors’ instruction
   1. Strongly agree
   2. Agree
   3. Disagree
   4. Strongly disagree
9. I am willing to quit smoking
   1. Strongly agree
   2. Agree
   3. Disagree
   4. Strongly disagree
10. I am willing to insist on taking medicine
    1. Strongly agree
    2. Agree
    3. Disagree
    4. Strongly disagree

**Questionnaire 3: Patients’ acceptance of smartphone health technology for hypertension management scale**

1. I often use mobile apps
   1. Strongly agree
   2. Agree
   3. Neutral
   4. Disagree
   5. Strongly disagree
2. I use smartphone to search health information on the Web
   1. Strongly agree
   2. Agree
   3. Neutral
   4. Disagree
   5. Strongly disagree
3. I use mobile apps to help with managing health issues
   1. Strongly agree
   2. Agree
   3. Neutral
   4. Disagree
   5. Strongly disagree
4. Learning how to use mobile apps for disease management would be easy for me
   1. Strongly agree
   2. Agree
   3. Neutral
   4. Disagree
   5. Strongly disagree
5. Using mHealth apps in daily life is not troublesome
   1. Strongly agree
   2. Agree
   3. Neutral
   4. Disagree
   5. Strongly disagree
6. The content of mHealth apps is easy to understand
   1. Strongly agree
   2. Agree
   3. Neutral
   4. Disagree
   5. Strongly disagree
7. I can do what I want to do easily by using mHealth apps
   1. Strongly agree
   2. Agree
   3. Neutral
   4. Disagree
   5. Strongly disagree
8. Knowing that a doctor checks my blood pressure data gives me confidence in hypertension management
   1. Strongly agree
   2. Agree
   3. Neutral
   4. Disagree
   5. Strongly disagree
9. Logging or sending blood pressure values would make me cope with hypertension better
   1. Strongly agree
   2. Agree
   3. Neutral
   4. Disagree
   5. Strongly disagree
10. I would keep using mHealth apps for hypertension management
    1. Strongly agree
    2. Agree
    3. Neutral
    4. Disagree
    5. Strongly disagree
