# Supplementary material for: Using Goal-Directed Design to Create a Mobile Health App to Improve Patient Compliance With Hypertension Self-Management: Development and Deployment
Source: JMIR Mhealth Uhealth. 2020 Feb 25;8(2):e14466. doi: 10.2196/14466 (PMC7064970; doi:10.2196/14466)
Supplement: Multimedia Appendix 2 [file mhealth_v8i2e14466_app2.docx]

**An overview of the articles and apps found in Stage 2**

To investigate which functional modules have previously been included in existing mHealth apps, we searched for literature on PubMed and searched for apps and downloaded them from the app store. A total of 36 references and 37 apps were screened and analyzed. The concrete lists are provided as below. The results of investigating functional modules in existing mHealth apps is shown in Table 1. These results demonstrated that a variety of functional modules designed in existing mHealth apps focus primarily on self-monitoring. Only some of the apps considered supervision and intervention from doctors. We proposed our functional designs based on the investigation results.

Table 1. Investigation results of functional modules in existing mHealth apps

| **Category** | **Functional Module** | | **References**  **n=36** | **Apps**  **n=37** | **Total**  **n=73** |
| --- | --- | --- | --- | --- | --- |
|  |  | |  |  |  |
| **Early detection** | Risk assessment | | 5 | 16 | 21 |
| **Disease cognition** | Health education | | 13 | 23 | 36 |
| **Lifestyle intervention** | Recipes | | 1 | 11 | 12 |
|  | Exercise Plan | | 2 | 3 | 5 |
| **Disease management and control** | Self-monitoring | BP / blood glucose | 36 | 37 | 73 |
|  |  | Weight | 27 | 21 | 48 |
|  |  | Medication | 12 | 35 | 47 |
|  |  | Diet | 8 | 14 | 22 |
|  |  | Exercise | 8 | 12 | 20 |
|  | Statistical report | | 11 | 20 | 31 |
|  | Reminder service | | 5 | 18 | 23 |
|  | Abnormal warning | | 8 | 4 | 12 |
| **Intervention of doctors** | Short message service | | 12 | 7 | 19 |
|  | Telephone follow-up | | 6 | 4 | 10 |
|  | Online consultation | | 10 | 8 | 18 |
| **Gamification design** | Challenge and reward | | 4 | 0 | 4 |
|  | Leaderboard | | 3 | 0 | 3 |
|  | Social contact | | 5 | 10 | 15 |

**The screened literature list in Stage 2**

﻿1. Varleta P, Acevedo M, Akel C, et al. Mobile phone text messaging improves antihypertensive drug adherence in the community. J Clin Hypertens (Greenwich). 2017;19(12):1276-1284.

﻿2. Ajay VS, Jindal D, Roy A, et al. Development of a Smartphone-Enabled Hypertension and Diabetes Mellitus Management Package to Facilitate Evidence-Based Care Delivery in Primary Healthcare Facilities in India: The mPower Heart Project. J Am Heart Assoc. 2016;5(12).

﻿3. Bengtsson U, Kjellgren K, Hallberg I, Lindwall M, Taft C. Improved Blood Pressure Control Using an Interactive Mobile Phone Support System. J Clin Hypertens (Greenwich). 2016;18(2):101-108.

﻿4. Bobrow K, Farmer AJ, Springer D, et al. Mobile Phone Text Messages to Support Treatment Adherence in Adults With High Blood Pressure (SMS-Text Adherence Support [StAR]): A Single-Blind, Randomized Trial. Circulation. 2016;133(6):592-600.

﻿5. Kang H, Park H-A. A Mobile App for Hypertension Management Based on Clinical Practice Guidelines: Development and Deployment. JMIR mHealth uHealth. 2016;4(1):e12.

﻿6. Maslakpak MH, Safaie M. A Comparison between The Effectiveness of Short Message Service and Reminder Cards Regarding Medication Adherence in Patients with Hypertension: A Randomized Controlled Clinical Trial. Int J community based Nurs midwifery. 2016;4(3):209-218.

﻿7. Davidson TM, McGillicuddy J, Mueller M, et al. Evaluation of an mHealth Medication Regimen Self-Management Program for African American and Hispanic Uncontrolled Hypertensives. J Pers Med. 2015;5(4):389-405.

﻿8. Golshahi J, Ahmadzadeh H, Sadeghi M, Mohammadifard N, Pourmoghaddas A. Effect of self-care education on lifestyle modification, medication adherence and blood pressure in hypertensive adults: Randomized controlled clinical trial. Adv Biomed Res. 2015;4:204.

﻿9. Kamal AK, Shaikh Q, Pasha O, et al. A randomized controlled behavioral intervention trial to improve medication adherence in adult stroke patients with prescription tailored Short Messaging Service (SMS)-SMS4Stroke study. BMC Neurol. 2015;15:212.

﻿10. Khonsari S, Subramanian P, Chinna K, Latif LA, Ling LW, Gholami O. Effect of a reminder system using an automated short message service on medication adherence following acute coronary syndrome. Eur J Cardiovasc Nurs. 2015;14(2):170-179.

﻿11. Arora S, Peters AL, Burner E, Lam CN, Menchine M. Trial to examine text message-based mHealth in emergency department patients with diabetes (TExT-MED): a randomized controlled trial. Ann Emerg Med. 2014;63(6):745-54.e6.

﻿12. Piette JD, Valverde H, Marinec N, et al. Establishing an independent mobile health program for chronic disease self-management support in bolivia. Front public Heal. 2014;2:95.

﻿13. Brath H, Morak J, Kästenbauer T, et al. Mobile health (mHealth) based medication adherence measurement - a pilot trial using electronic blisters in diabetes patients. Br J Clin Pharmacol. 2013;76 Suppl 1:47-55.

﻿14. Kirwan M, Vandelanotte C, Fenning A, Duncan MJ. Diabetes self-management smartphone application for adults with type 1 diabetes: randomized controlled trial. J Med Internet Res. 2013;15(11):e235.

﻿15. McGillicuddy JW, Gregoski MJ, Weiland AK, et al. Mobile Health Medication Adherence and Blood Pressure Control in Renal Transplant Recipients: A Proof-of-Concept Randomized Controlled Trial. JMIR Res Protoc. 2013;2(2):e32.

﻿16. Nagrebetsky A, Larsen M, Craven A, et al. Stepwise self-titration of oral glucose-lowering medication using a mobile telephone-based telehealth platform in type 2 diabetes: a feasibility trial in primary care. J Diabetes Sci Technol. 2013;7(1):123-134.

﻿17. Osborn CY, Mulvaney SA. Development and feasibility of a text messaging and interactive voice response intervention for low-income, diverse adults with type 2 diabetes mellitus. J Diabetes Sci Technol. 2013;7(3):612-622.

﻿18. Patel S, Jacobus-Kantor L, Marshall L, et al. Mobilizing your medications: an automated medication reminder application for mobile phones and hypertension medication adherence in a high-risk urban population. J Diabetes Sci Technol. 2013;7(3):630-639.

﻿19. Logan AG, Irvine MJ, McIsaac WJ, et al. Effect of home blood pressure telemonitoring with self-care support on uncontrolled systolic hypertension in diabetics. Hypertens (Dallas, Tex 1979). 2012;60(1):51-57.

﻿20. Migneault JP, Dedier JJ, Wright JA, et al. A culturally adapted telecommunication system to improve physical activity, diet quality, and medication adherence among hypertensive African-Americans: a randomized controlled trial. Ann Behav Med. 2012;43(1):62-73.

﻿21. Ryan D, Price D, Musgrave SD, et al. Clinical and cost effectiveness of mobile phone supported self-monitoring of asthma: multicentre randomised controlled trial. BMJ. 2012;344:e1756.

﻿22. Seto E, Leonard KJ, Cafazzo JA, Barnsley J, Masino C, Ross HJ. Mobile phone-based telemonitoring for heart failure management: a randomized controlled trial. J Med Internet Res. 2012;14(1):e31.

﻿23. Koehler F, Winkler S, Schieber M, et al. Impact of remote telemedical management on mortality and hospitalizations in ambulatory patients with chronic heart failure: the telemedical interventional monitoring in heart failure study. Circulation. 2011;123(17):1873-1880.

﻿24. Lim S, Kang SM, Shin H, et al. Improved glycemic control without hypoglycemia in elderly diabetic patients using the ubiquitous healthcare service, a new medical information system. Diabetes Care. 2011;34(2):308-313. doi:10.2337/dc10-1447

﻿25. Neumann CL, Menne J, Rieken EM, et al. Blood pressure telemonitoring is useful to achieve blood pressure control in inadequately treated patients with arterial hypertension. J Hum Hypertens. 2011;25(12):732-738.

﻿26. Quinn CC, Shardell MD, Terrin ML, Barr EA, Ballew SH, Gruber-Baldini AL. Cluster-randomized trial of a mobile phone personalized behavioral intervention for blood glucose control. Diabetes Care. 2011;34(9):1934-1942.

﻿27. Shetty AS, Chamukuttan S, Nanditha A, Raj RKC, Ramachandran A. Reinforcement of adherence to prescription recommendations in Asian Indian diabetes patients using short message service (SMS)--a pilot study. J Assoc Physicians India. 2011;59:711-714.

﻿28. Koehler F, Winkler S, Schieber M, et al. Impact of remote telemedical management on mortality and hospitalizations in ambulatory patients with chronic heart failure: the telemedical interventional monitoring in heart failure study. Circulation. 2011;123(17):1873-1880.

﻿29. Earle KA, Istepanian RSH, Zitouni K, Sungoor A, Tang B. Mobile telemonitoring for achieving tighter targets of blood pressure control in patients with complicated diabetes: a pilot study. Diabetes Technol Ther. 2010;12(7):575-579.

﻿30. Rossi MCE, Nicolucci A, Di Bartolo P, et al. Diabetes Interactive Diary: a new telemedicine system enabling flexible diet and insulin therapy while improving quality of life: an open-label, international, multicenter, randomized study. Diabetes Care. 2010;33(1):109-115.

﻿31. Prabhakaran L, Chee WY, Chua KC, Abisheganaden J, Wong WM. The use of text messaging to improve asthma control: a pilot study using the mobile phone short messaging service (SMS). J Telemed Telecare. 2010;16(5):286-290.

﻿32. Istepanian RSH, Sungoor A, Earle KA. Technical and compliance considerations for mobile health self-monitoring of glucose and blood pressure for patients with diabetes. Conf Proc . Annu Int Conf IEEE Eng Med Biol Soc IEEE Eng Med Biol Soc Annu Conf. 2009;2009:5130-5133.

﻿33. Istepanian RSH, Zitouni K, Harry D, et al. Evaluation of a mobile phone telemonitoring system for glycaemic control in patients with diabetes. J Telemed Telecare. 2009;15(3):125-128.

﻿34. Faridi Z, Liberti L, Shuval K, Northrup V, Ali A, Katz DL. Evaluating the impact of mobile telephone technology on type 2 diabetic patients’ self-management: the NICHE pilot study. J Eval Clin Pract. 2008;14(3):465-469.

﻿35. Madsen LB, Kirkegaard P, Pedersen EB. Blood pressure control during telemonitoring of home blood pressure. A randomized controlled trial during 6 months. Blood Press. 2008;17(2):78-86.

﻿36. Franklin VL, Waller A, Pagliari C, Greene SA. A randomized controlled trial of Sweet Talk, a text-messaging system to support young people with diabetes. Diabet Med. 2006;23(12):1332-1338.

**The screened app list in Stage 2**

1. SmartBP (iOS)

<https://apps.apple.com/cn/app/%E8%A1%80%E5%8E%8B%E7%AE%A1%E7%90%86-smartbp/id519076558>

2. BP Manager (iOS)

<https://apps.apple.com/cn/app/%E8%A1%80%E5%8E%8B%E7%AE%A1%E5%AE%B6-%E9%AB%98%E8%A1%80%E5%8E%8B%E5%BF%85%E5%A4%87%E8%BD%AF%E4%BB%B6/id458537528>

3. More Health (iOS)

<https://apps.apple.com/cn/app/%E5%A6%99%E5%81%A5%E5%BA%B7/id841386224>

4. 365 Pulse and Blood Pressure (iOS)

<https://apps.apple.com/cn/app/365pulse-and-blood-pressure/id1469201203>

5. Hypertension Manager (iOS)

<https://apps.apple.com/cn/app/%E9%AB%98%E8%A1%80%E5%8E%8B%E7%AE%A1%E5%AE%B6/id929001721>

6. Hypertension Doctor (iOS)

<https://apps.apple.com/cn/app/%E9%AB%98%E8%A1%80%E5%8E%8B%E5%A4%A7%E5%A4%AB-%E6%82%A3%E8%80%85%E7%89%88-%E5%85%A8%E5%9B%BD%E9%AB%98%E8%A1%80%E5%8E%8B%E6%99%BA%E8%83%BD%E7%AE%A1%E7%90%86%E5%B9%B3%E5%8F%B0/id1181821858>

7. Hypertension Diet Wiki (iOS)

<https://apps.apple.com/cn/app/%E9%AB%98%E8%A1%80%E5%8E%8B%E5%85%BB%E7%94%9F%E9%A3%9F%E7%96%97%E7%99%BE%E7%A7%91/id992334958>

8. Wei Glucose (iOS)

<https://apps.apple.com/cn/app/%E5%BE%AE%E7%B3%96-%E7%B3%96%E5%B0%BF%E7%97%85%E8%A1%80%E7%B3%96%E5%81%A5%E5%BA%B7%E6%8E%A7%E7%B3%96%E5%87%8F%E8%82%A5%E9%99%8D%E4%B8%89%E9%AB%98%E5%88%A9%E5%99%A8/id725198656>

9. Control Diabetes (iOS)

<https://apps.apple.com/cn/app/%E6%8E%8C%E6%8E%A7%E7%B3%96%E5%B0%BF%E7%97%85-%E4%B8%93%E4%B8%9A%E7%9A%84%E6%99%BA%E8%83%BD%E7%B3%96%E5%B0%BF%E7%97%85%E5%85%A8%E7%A8%8B%E7%AE%A1%E7%90%86%E5%B9%B3%E5%8F%B0/id724054818>

10. Tang Shi (iOS)

<https://apps.apple.com/cn/app/%E7%B3%96%E5%A3%AB-%E4%B8%93%E4%B8%9A%E7%B3%96%E5%B0%BF%E7%97%85%E5%86%85%E5%AE%B9%E7%A4%BE%E5%8C%BA/id1218105131>

11. Glucose Nurse (iOS)

<https://apps.apple.com/cn/app/%E7%B3%96%E6%8A%A4%E5%A3%AB-%E7%B3%96%E5%B0%BF%E7%97%85%E7%AE%A1%E7%90%86%E6%B5%8B%E8%A1%80%E7%B3%96%E5%8A%A9%E6%89%8B/id698015255>

12. Diabetes Diet Wiki (iOS)

<https://apps.apple.com/cn/app/%E7%B3%96%E5%B0%BF%E7%97%85%E5%85%BB%E7%94%9F%E9%A3%9F%E7%96%97%E7%99%BE%E7%A7%91/id992334954>

13. Wen Glucose (iOS)

<https://apps.apple.com/cn/app/%E7%A8%B3%E7%B3%96-1%E4%BA%BF%E7%B3%96%E5%8F%8B%E7%9A%84%E9%80%89%E6%8B%A9-%E7%B3%96%E5%B0%BF%E7%97%85%E4%BA%BA%E5%A5%BD%E5%B8%AE%E6%89%8B/id1138308689>

14. Tang Tang Quan (iOS)

<https://apps.apple.com/cn/app/%E7%B3%96%E7%B3%96%E5%9C%88-%E8%AE%A91%E5%9E%8B%E7%B3%96%E5%B0%BF%E7%97%85%E6%9B%B4%E6%9C%89%E4%B9%90%E8%B6%A3/id1053422217>

15. Yi Glucose (iOS)

<https://apps.apple.com/cn/app/%E5%AE%9C%E7%B3%96-%E7%B3%96%E5%B0%BF%E7%97%85%E5%BF%85%E5%A4%87/id1177253446>

16. One Drop (iOS)

<https://apps.apple.com/cn/app/one-drop-%E7%B3%96%E5%B0%BF%E7%97%85%E7%AE%A1%E7%90%86/id972238816>

17. Diabetes Doctor (iOS)

<https://apps.apple.com/cn/app/%E7%B3%96%E5%8C%BB%E7%94%9F-%E7%B3%96%E5%B0%BF%E7%97%85%E5%81%A5%E5%BA%B7%E7%AE%A1%E7%90%86%E4%B8%93%E5%AE%B6/id923809458>

18. Tang Da Da (iOS)

<https://apps.apple.com/cn/app/%E7%B3%96%E5%A4%A7%E5%A4%A7-%E7%B3%96%E5%B0%BF%E7%97%85%E4%BA%A4%E6%B5%81%E5%B9%B3%E5%8F%B0/id942810308>

19. Sui Glucose (iOS)

<https://apps.apple.com/cn/app/%E9%9A%8F%E7%B3%96-%E7%B3%96%E5%B0%BF%E7%97%85%E7%B2%BE%E7%BB%86%E5%BC%BA%E5%8C%96%E7%AE%A1%E7%90%86/id1044415922>

20. Tang Bo Hu (iOS)

<https://apps.apple.com/cn/app/%E7%B3%96%E4%BC%AF%E8%99%8E/id1070836998>

21. Kang Kang Blood Pressure (Android)

<https://android.myapp.com/myapp/detail.htm?apkName=com.comoncare>

22. Yuyue Health Manager (Android)

<https://android.myapp.com/myapp/detail.htm?apkName=com.yuwell.uhealth>

23. Miao Health (Android)

<https://android.myapp.com/myapp/detail.htm?apkName=cn.funtalk.miao>

24. Wearfit (Android)

<https://android.myapp.com/myapp/detail.htm?apkName=com.wakeup.smartband>

25. Angel Doctor (Android)

<https://android.myapp.com/myapp/detail.htm?apkName=com.jumper.fhrinstruments>

26. WearHeart (Android)

<https://android.myapp.com/myapp/detail.htm?apkName=com.zjw.wearheart>

27. Ai Jia Kang (Android)

<https://android.myapp.com/myapp/detail.htm?apkName=iHealth.AiJiaKang.MI>

28. Tang Yi Bang

<https://android.myapp.com/myapp/detail.htm?apkName=com.app.npdc>

29. Diabetes (Android)

<https://android.myapp.com/myapp/detail.htm?apkName=anace.com.audiobooks.diabetes>

30. Zhang Shang Glucose Doctor (Android)

<https://android.myapp.com/myapp/detail.htm?apkName=cn.dreamplus.wentang>

31. Your Doctor (Android)

<https://android.myapp.com/myapp/detail.htm?apkName=com.yunio.hsdoctor>

32. Big Glucose Doctor (Android)

<https://android.myapp.com/myapp/detail.htm?apkName=com.threeti.huimapatient>

33. Hong Bei Xin (Android)

<https://android.myapp.com/myapp/detail.htm?apkName=com.yunio.heartsquare>

34. Tang Ba (Android)

<https://android.myapp.com/myapp/detail.htm?apkName=com.bianla.tangba>

35. HuaYi Glucose Manager (Android)

<https://android.myapp.com/myapp/detail.htm?apkName=sinomedisite.tmj.iglupadapp>

36. Yu Tang (Android)

<https://android.myapp.com/myapp/detail.htm?apkName=com.ihealth.chronos.patient.mi>

37. MeiQi Glucose Monitor (Android)

<https://android.myapp.com/myapp/detail.htm?apkName=com.bugull.meiqiguard>
