# Supplementary material for: Using Goal-Directed Design to Create a Mobile Health App to Improve Patient Compliance With Hypertension Self-Management: Development and Deployment
Source: JMIR Mhealth Uhealth. 2020 Feb 25;8(2):e14466. doi: 10.2196/14466 (PMC7064970; doi:10.2196/14466)
Supplement: Multimedia Appendix 3 [file mhealth_v8i2e14466_app3.pdf]

Management Plan Module

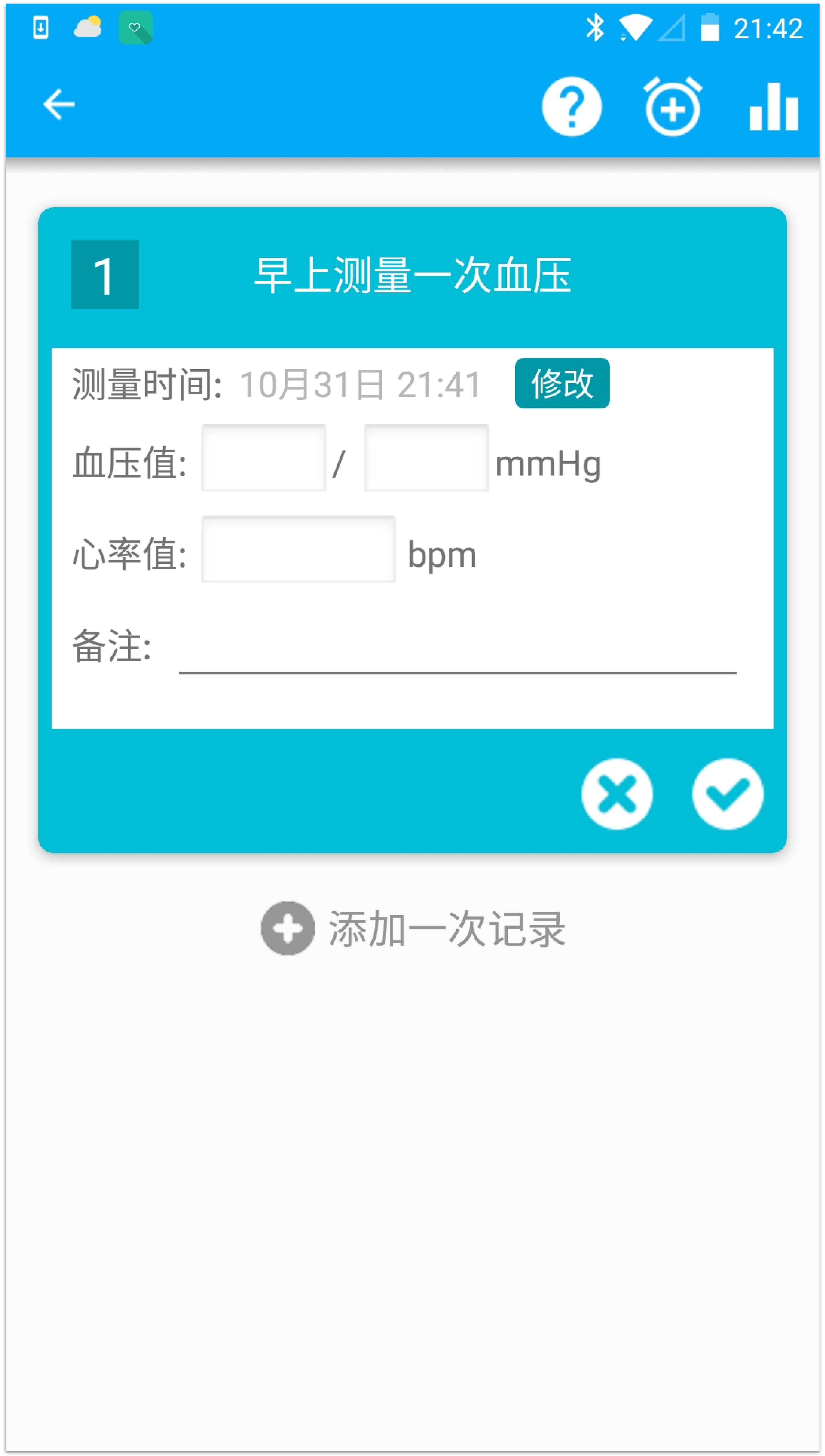

BP Record TODO Card

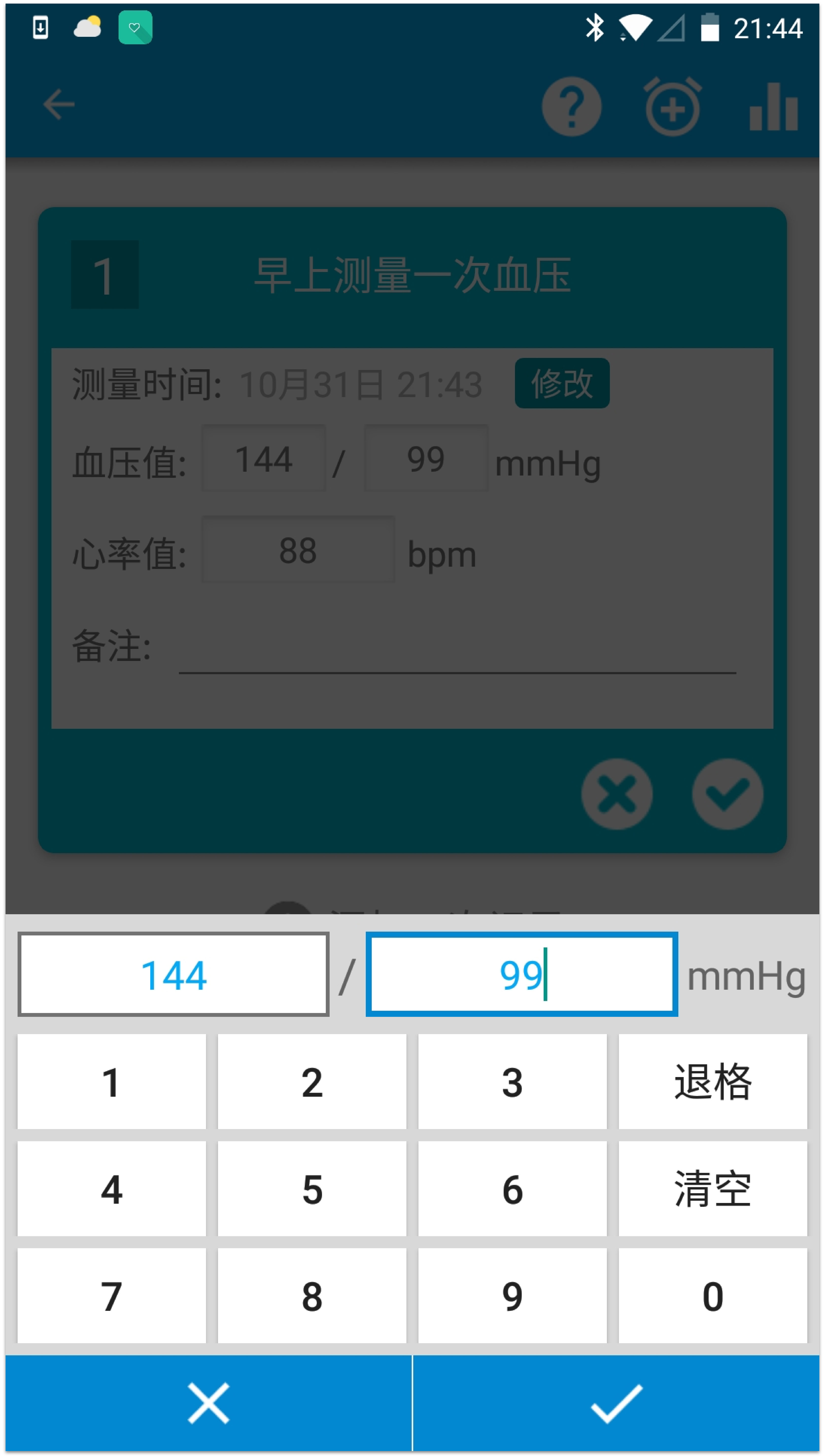

Input BP Data

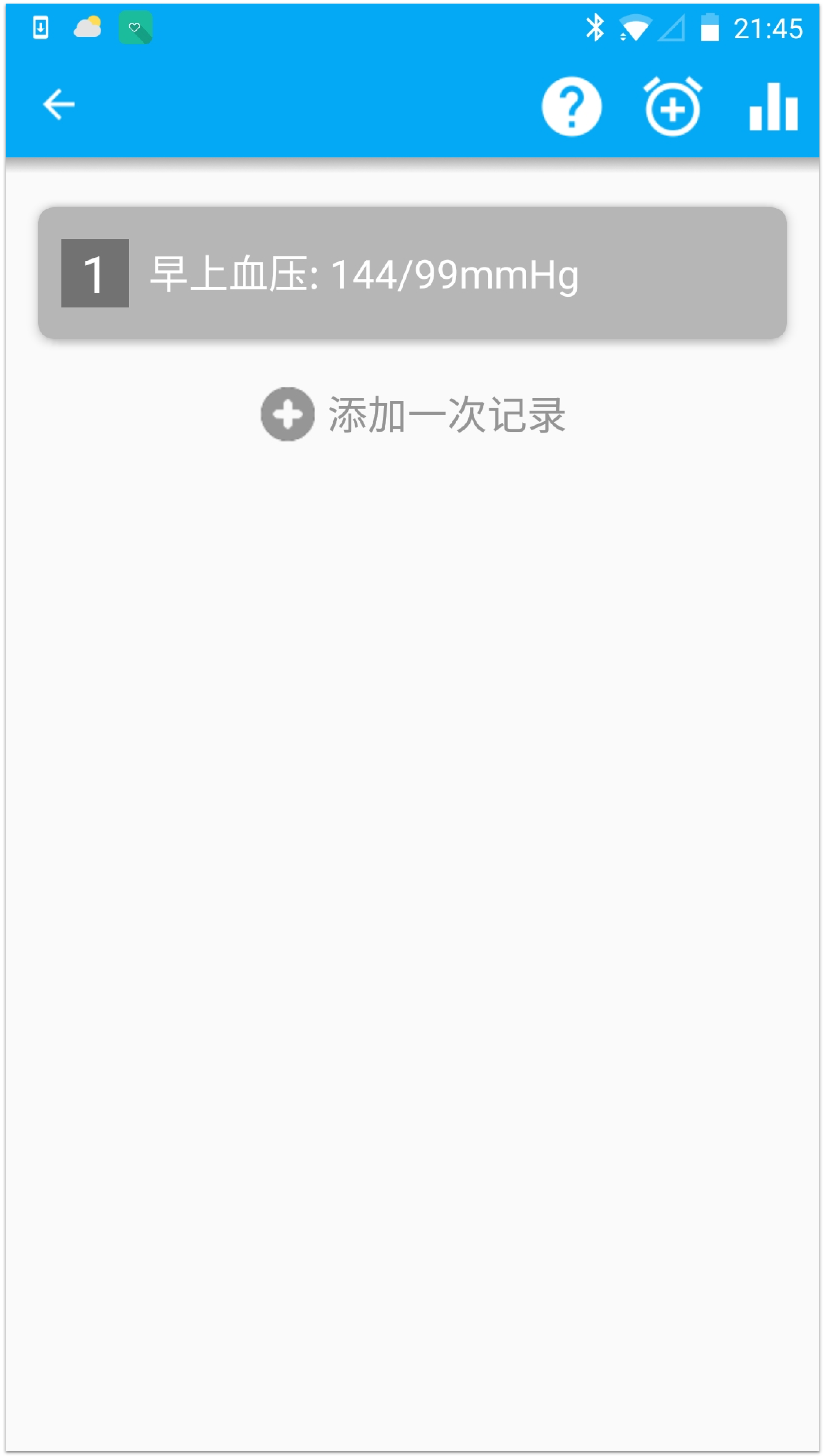

BP Data upload finished

Reminder Service Module

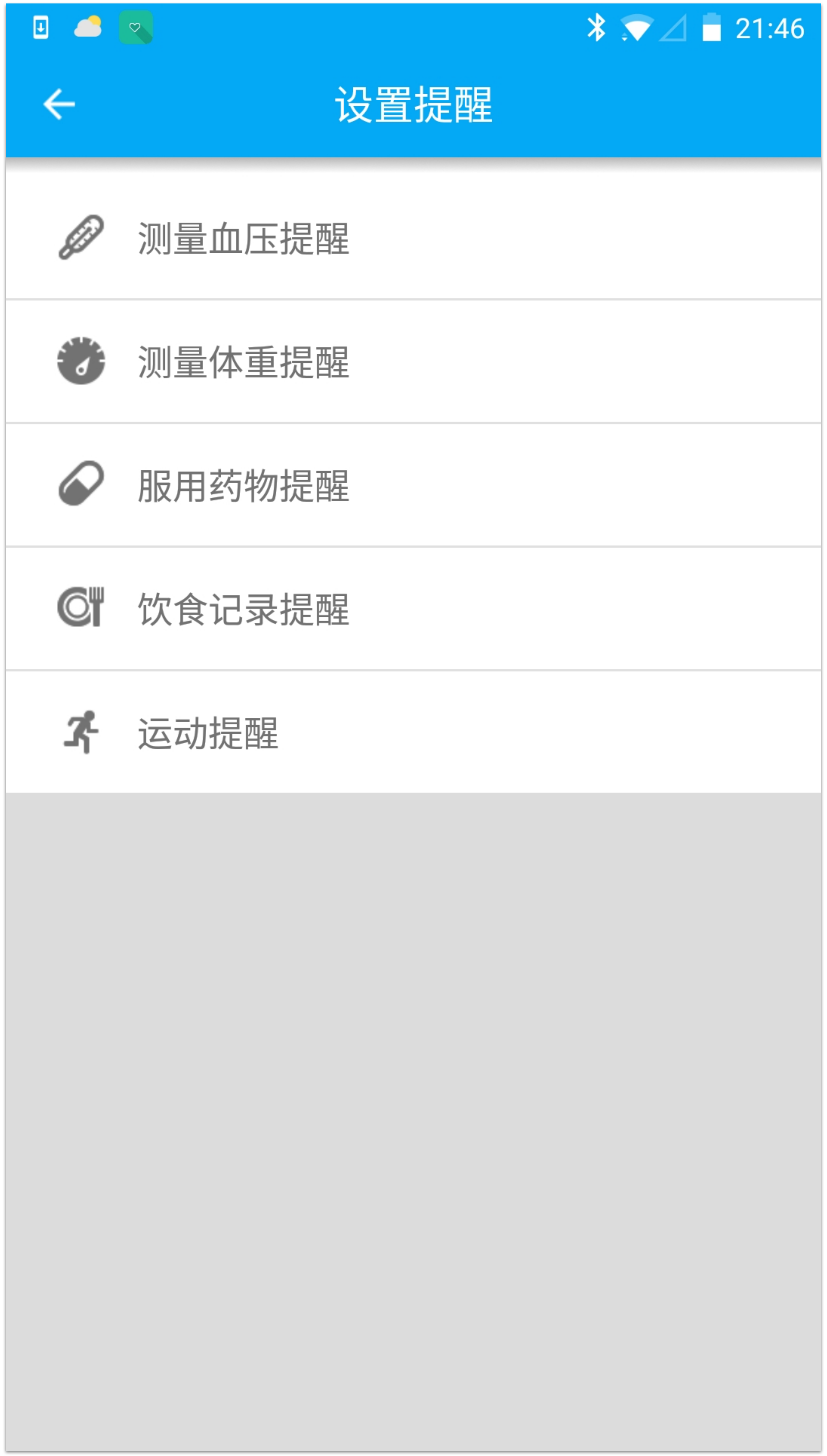

Reminder List

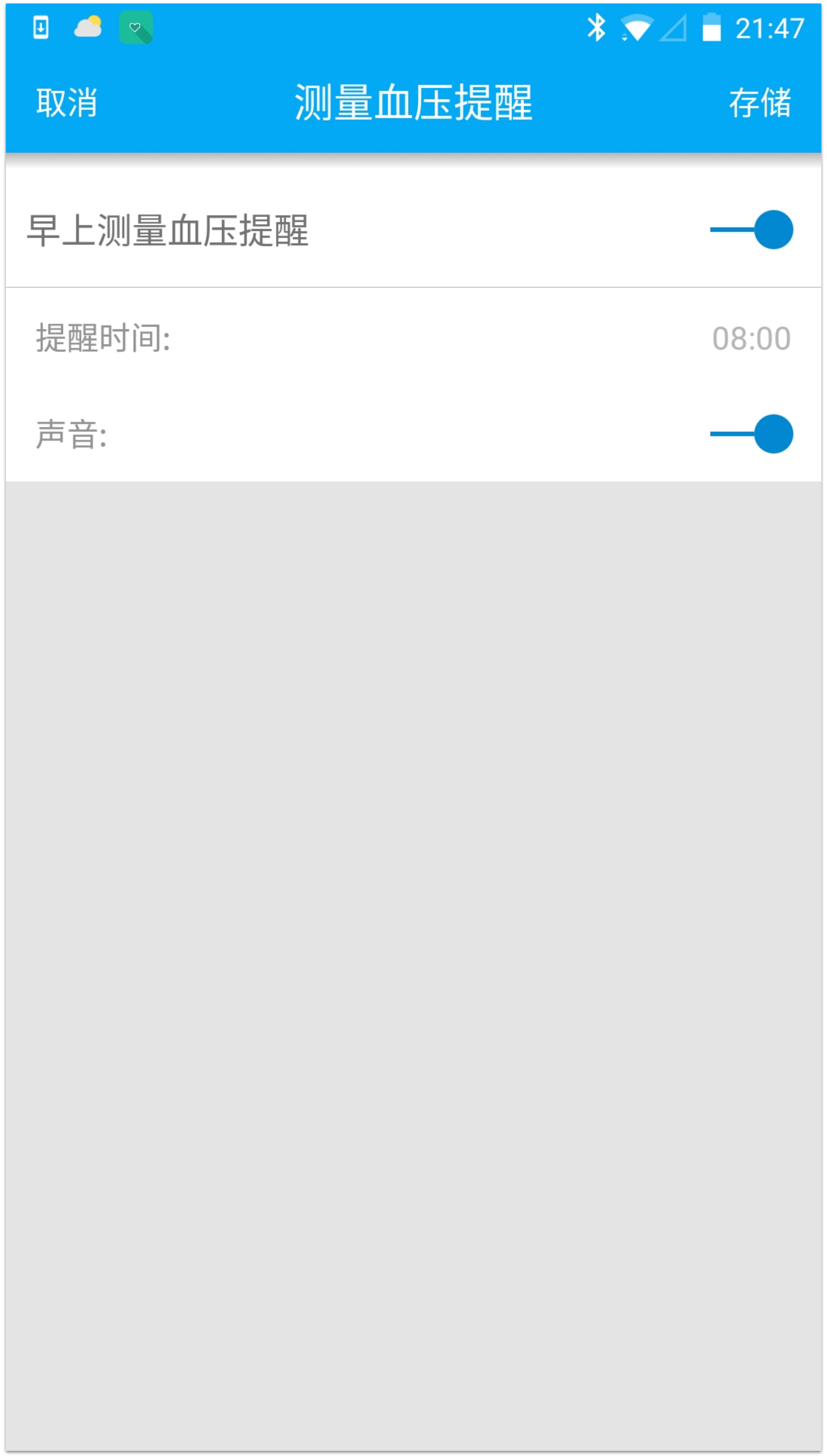

BP Measuring Reminder

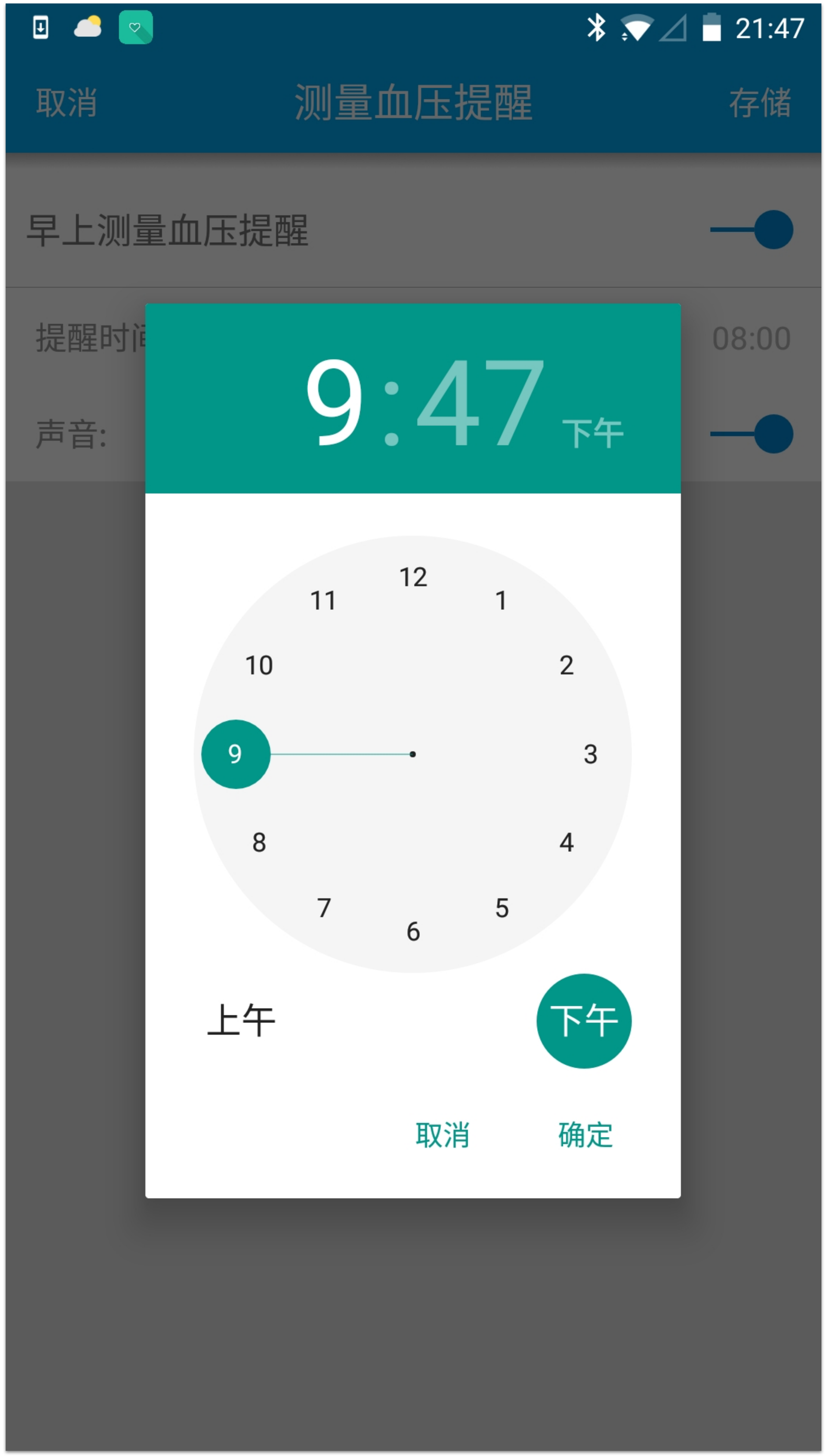

Modify Reminding Time

Health Report Module

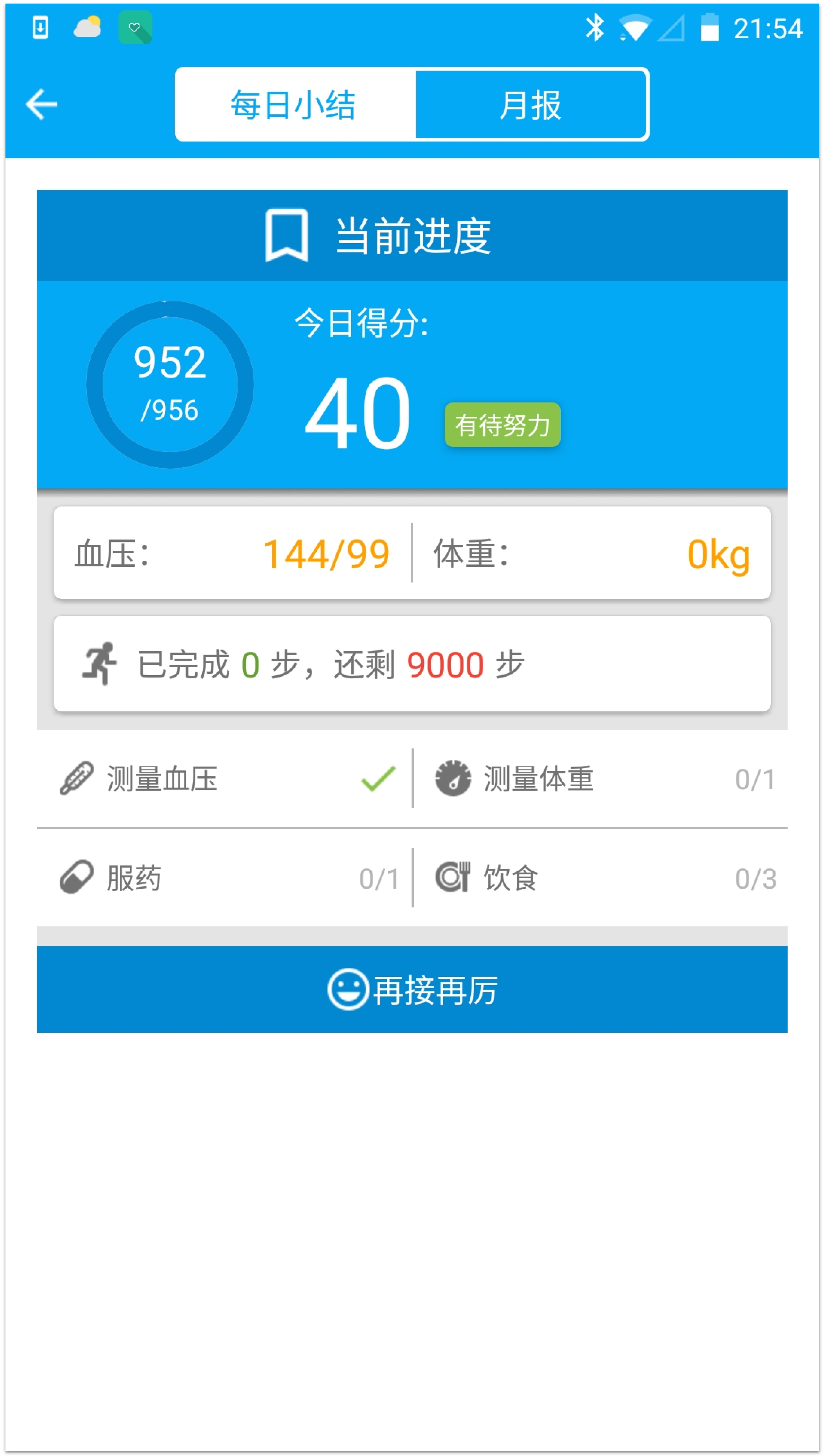

Health Daily Report

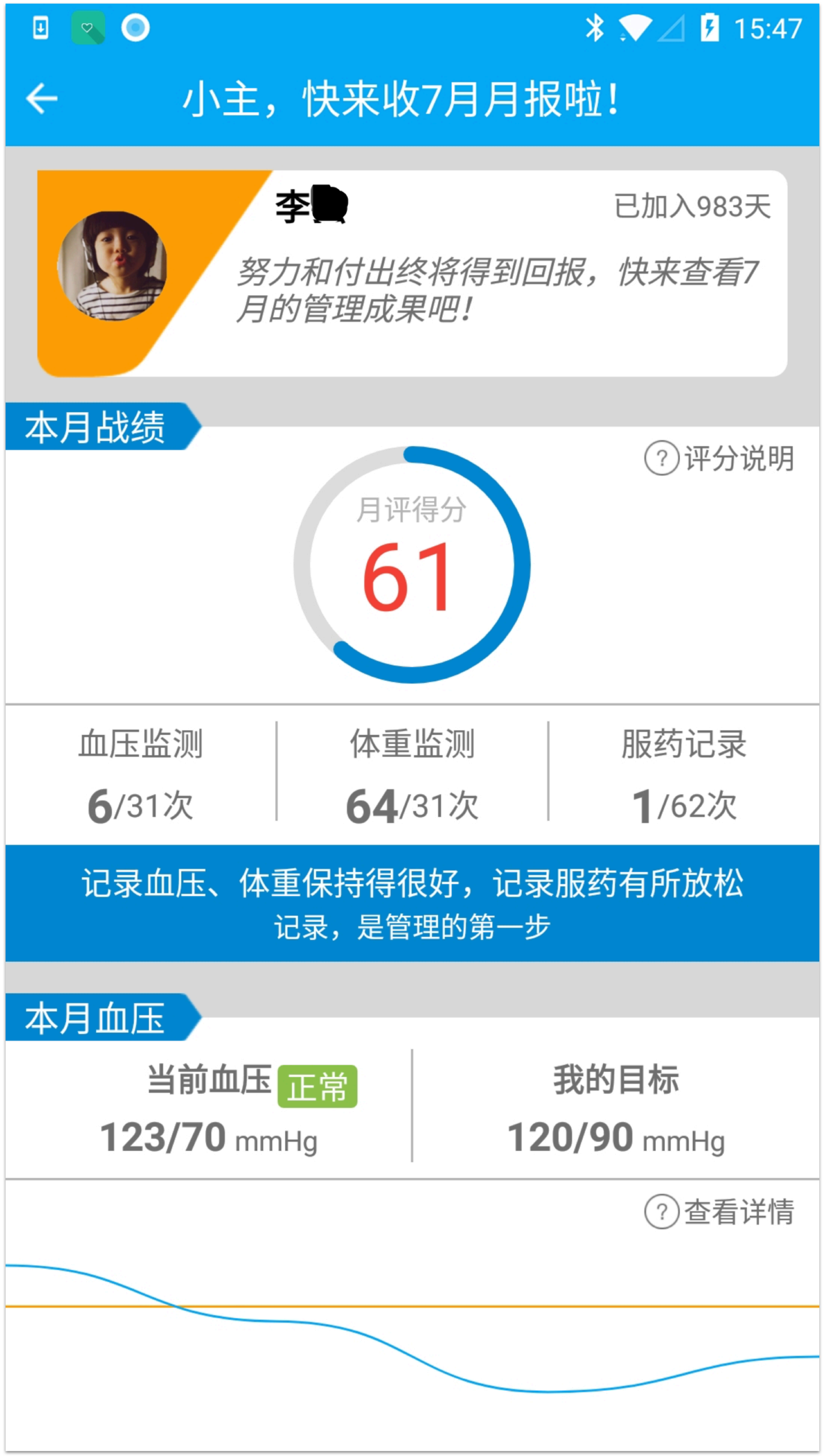

Health Monthly Report-part1

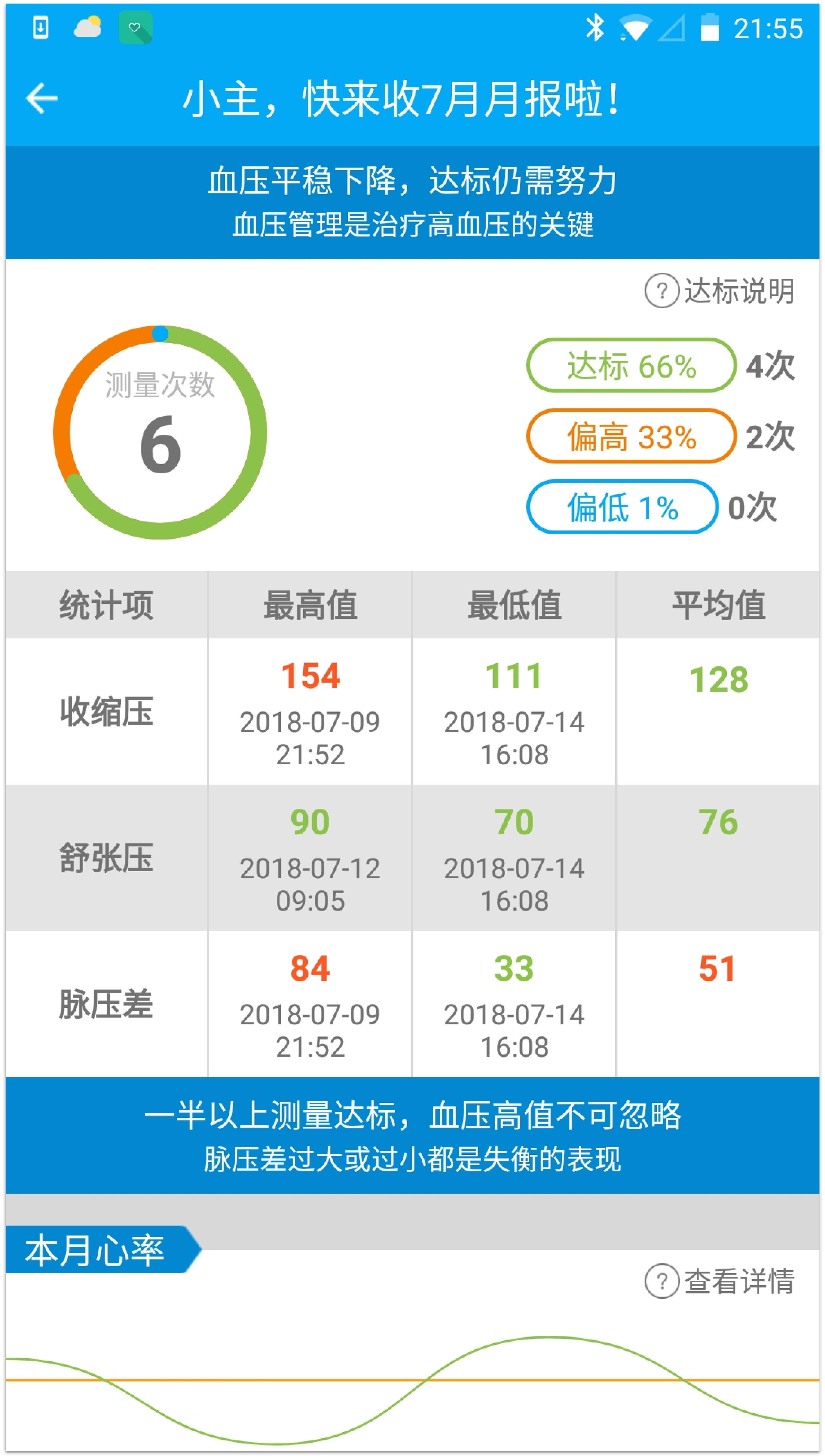

Health Monthly Report-part2

Leaderboard Module

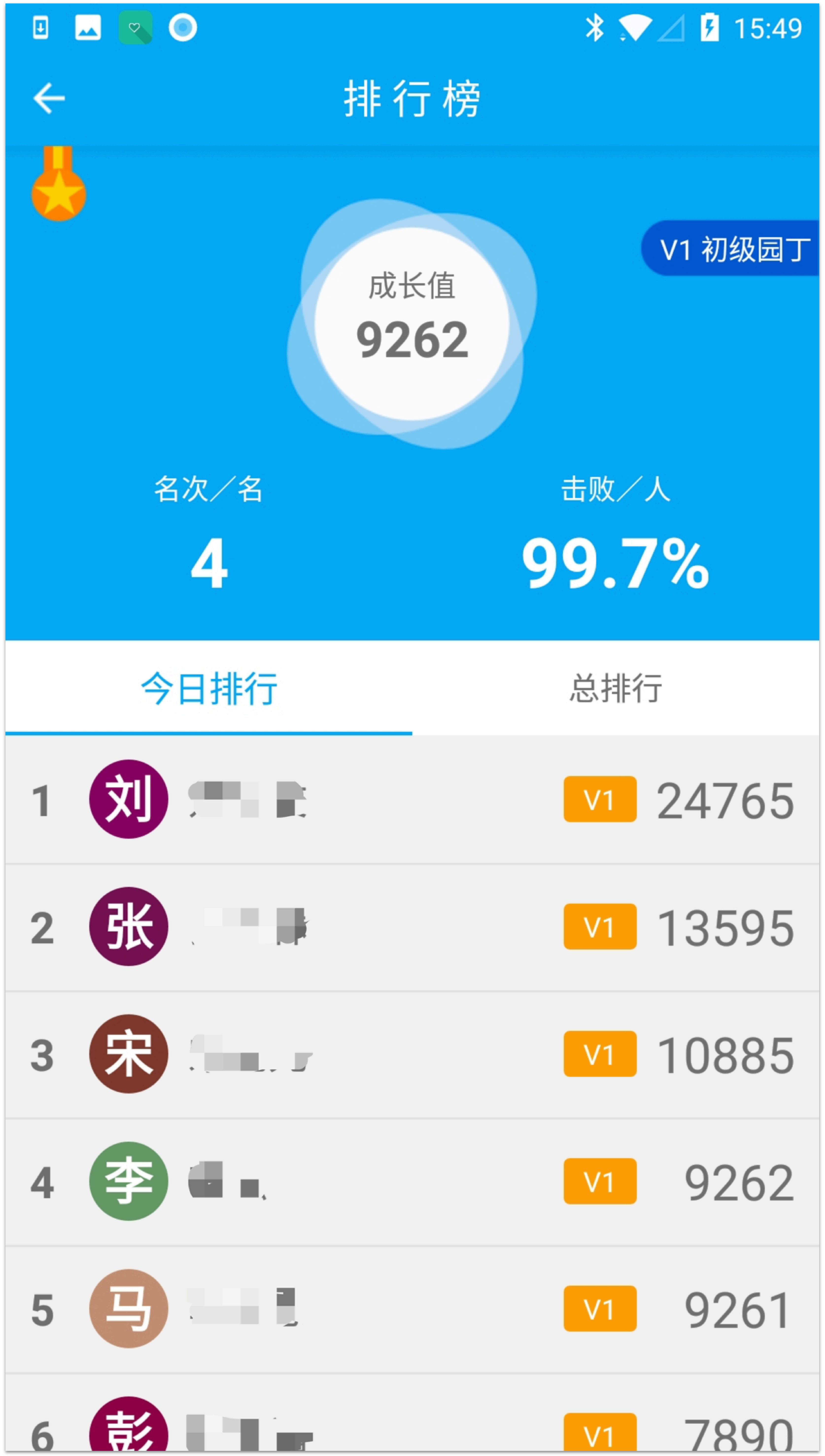

Today Leaderboard

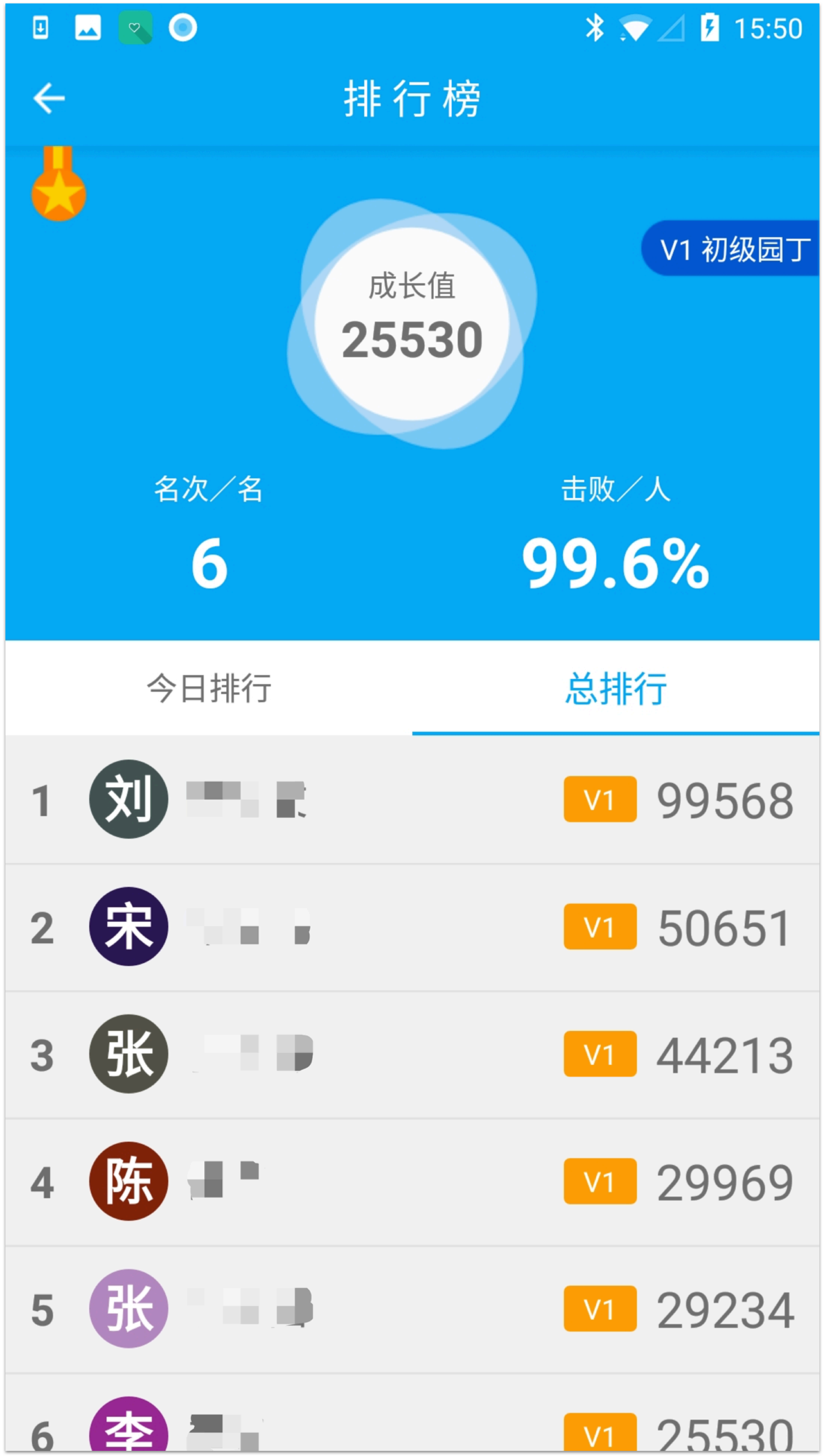

Total Leaderboard

Health Education Module

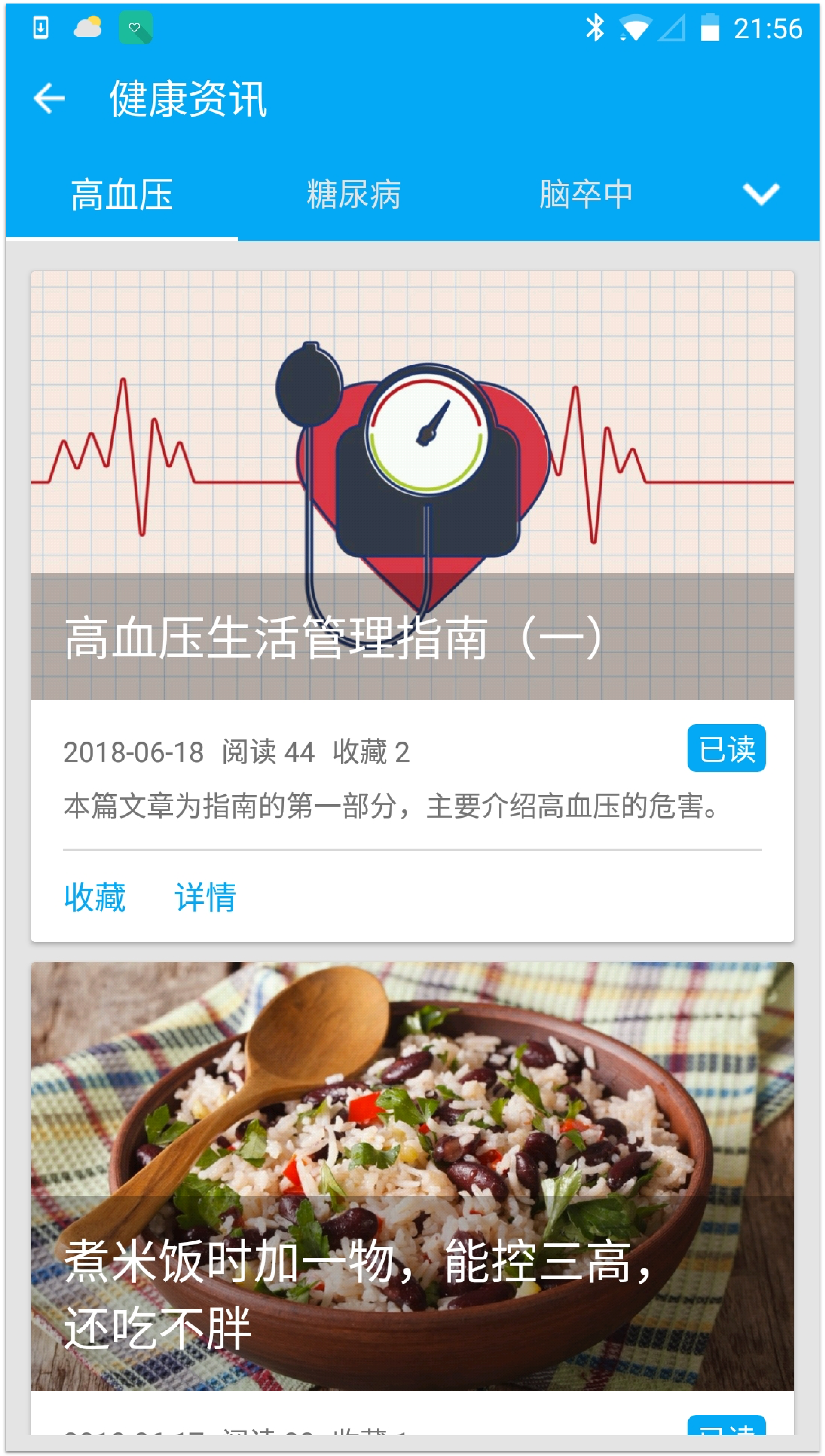

Health Knwledge List

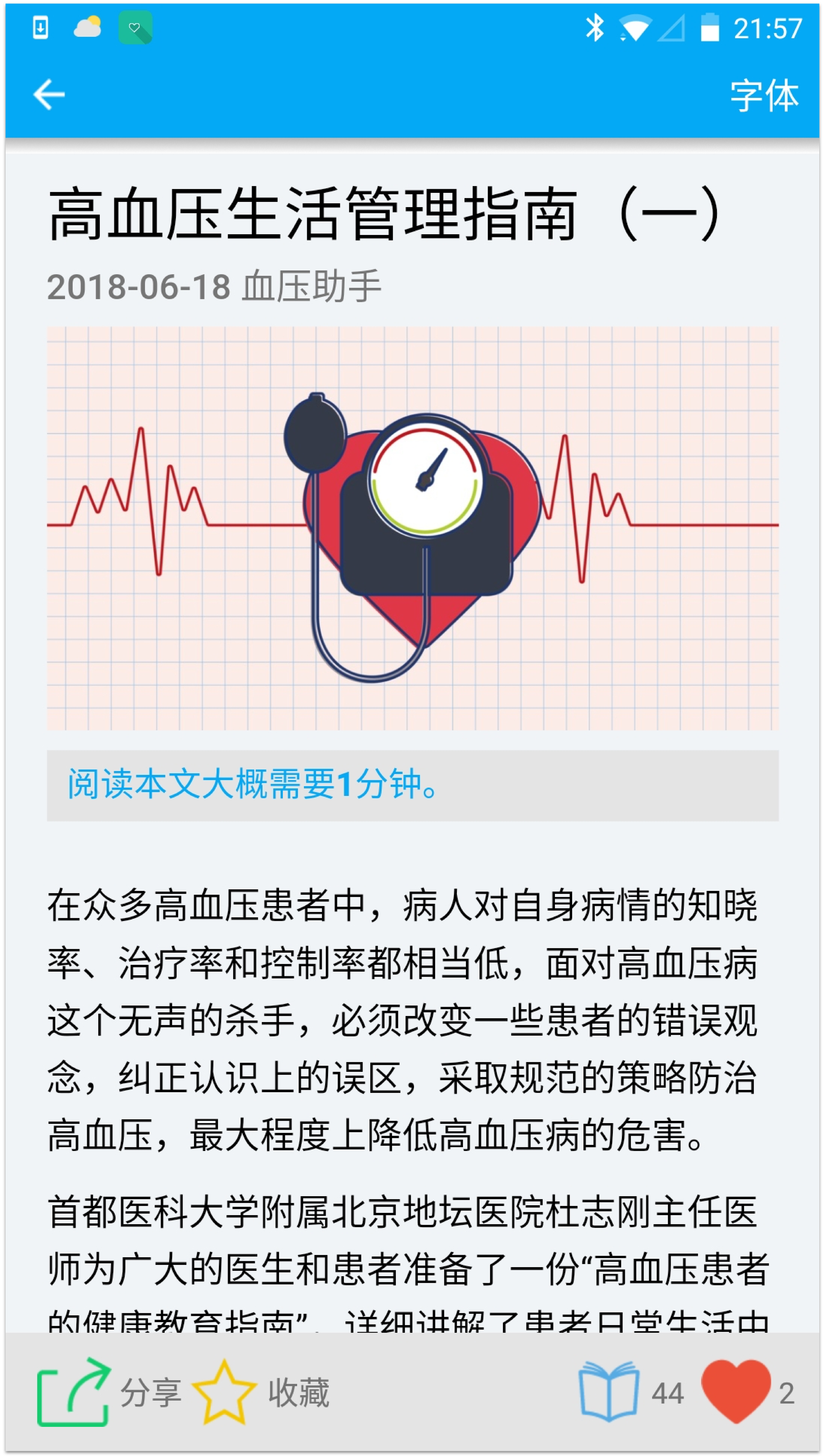

Read Health Knowledge

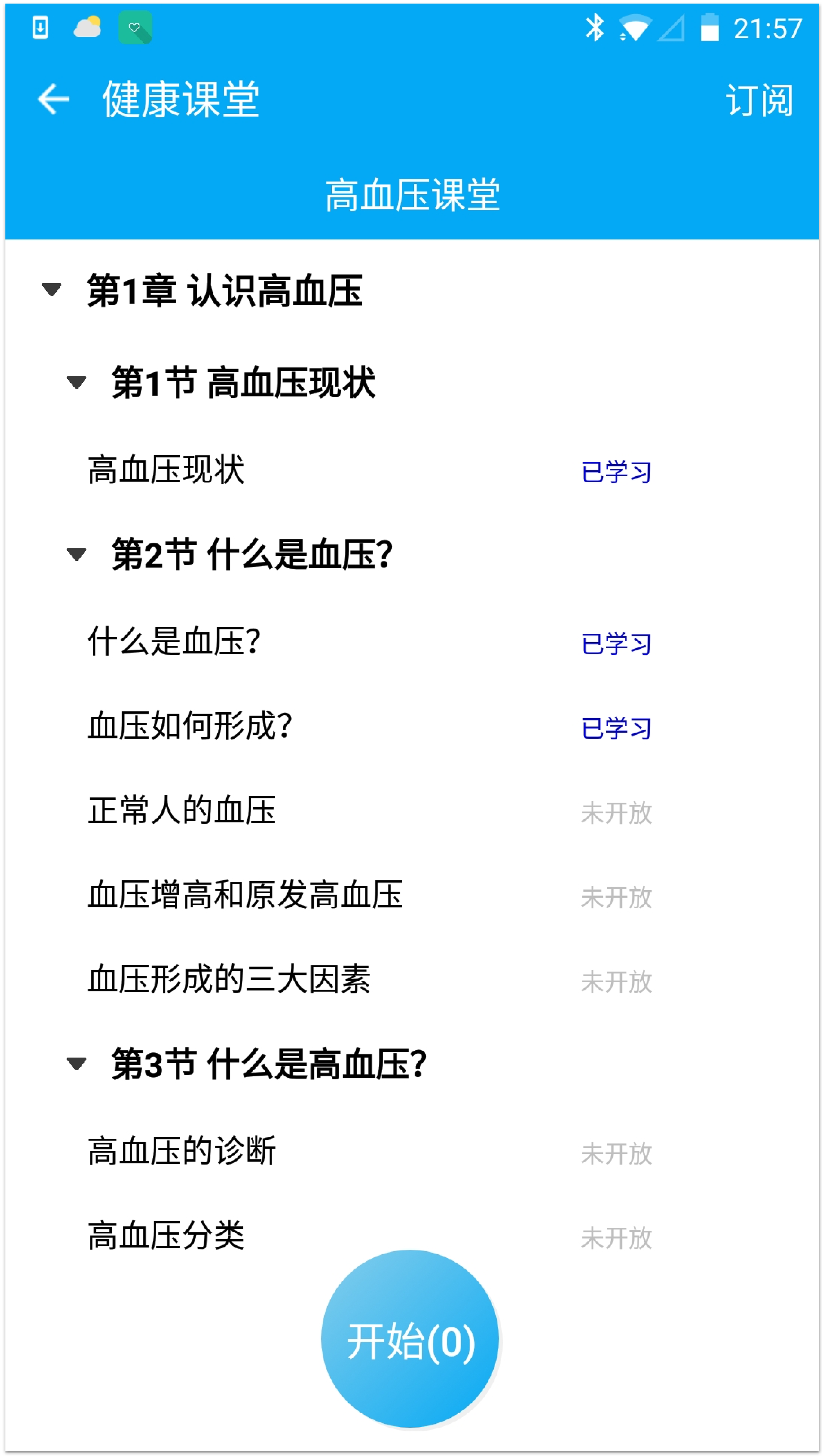

Systematic Health Education

Health Check-up Module

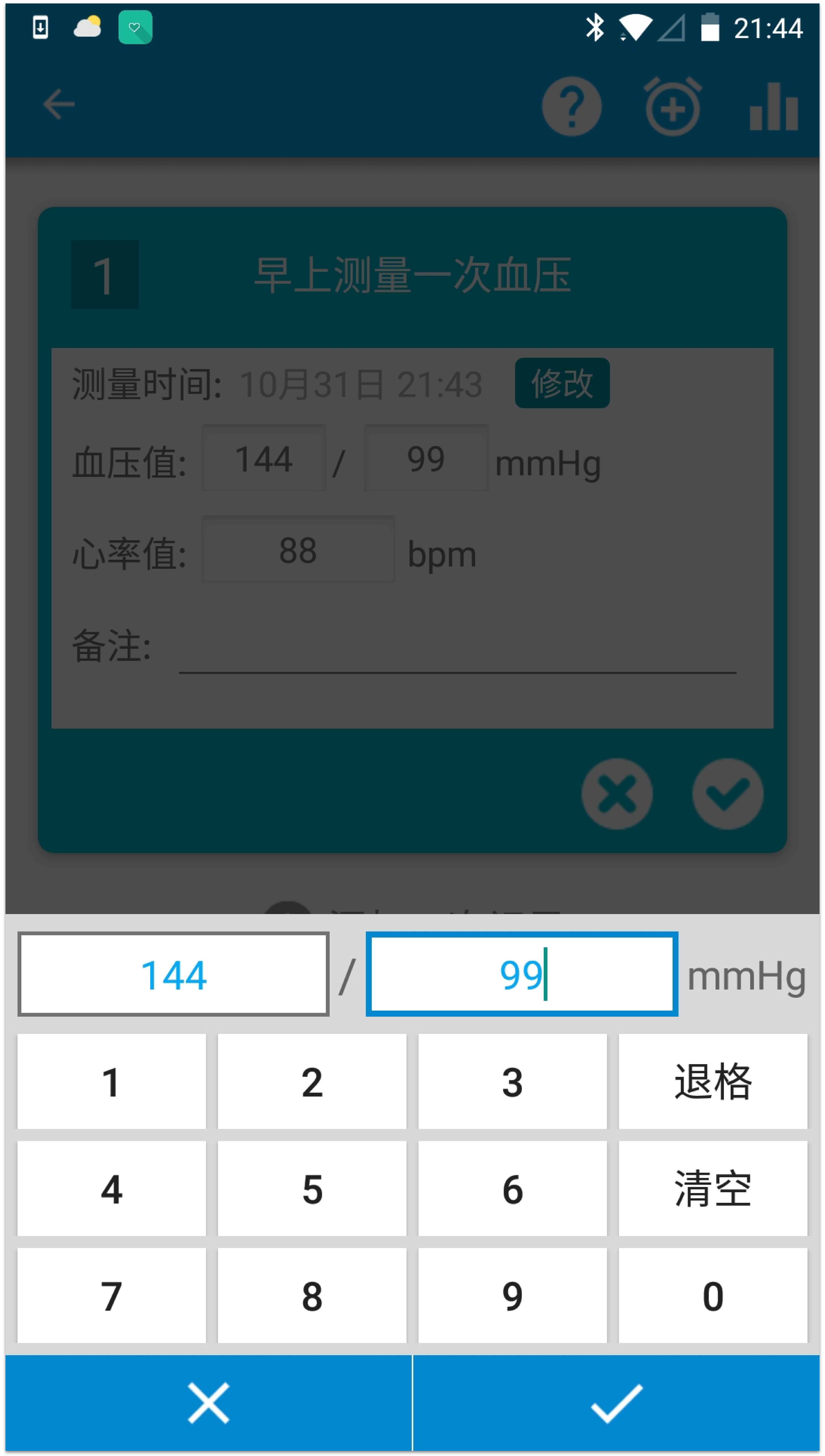

Input BP Data

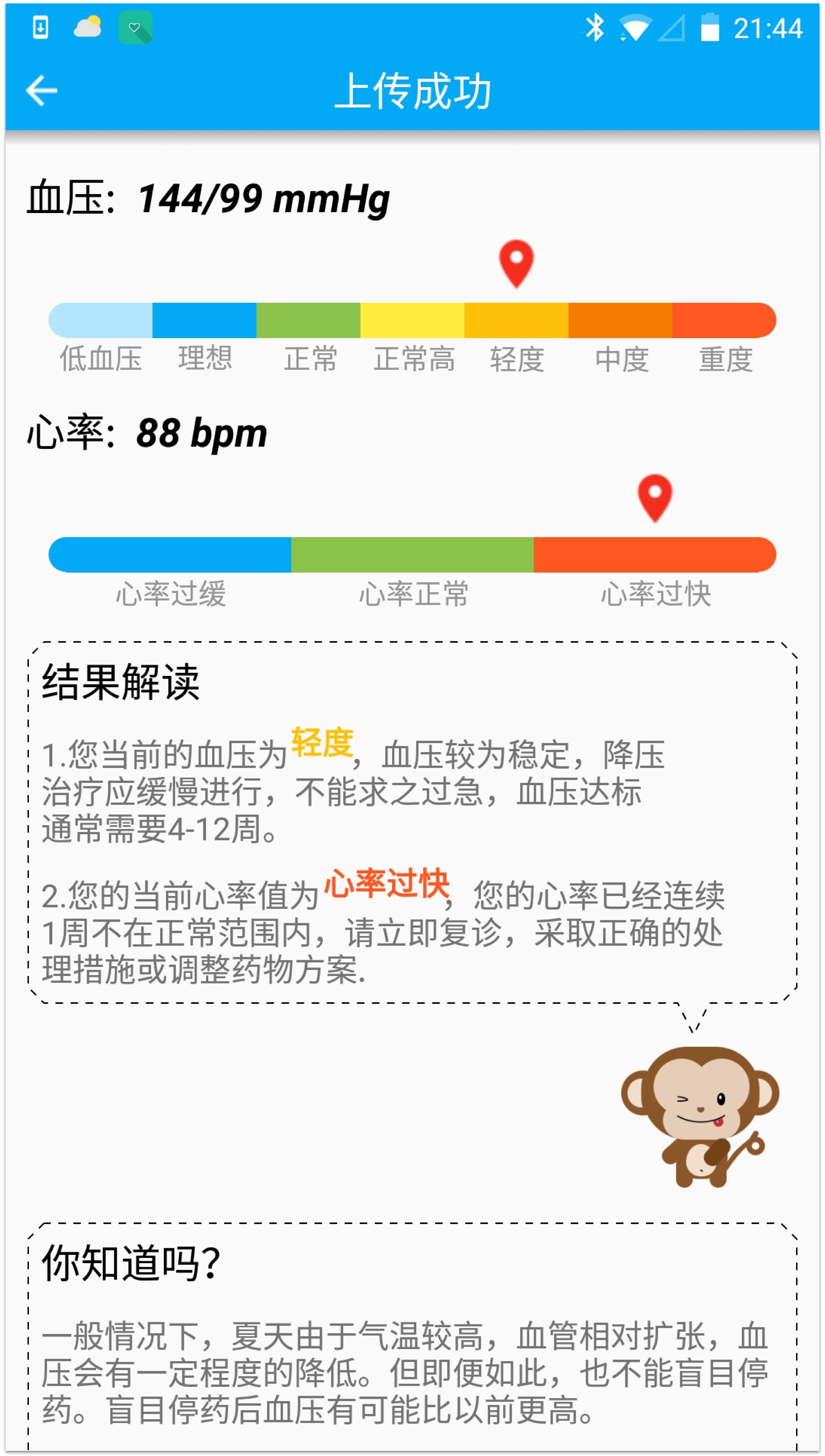

Intelligent Feedback of BP Data
